# Breast Cancer Treatments: Updates and New Challenges

**DOI:** 10.3390/jpm11080808

**Published:** 2021-08-19

**Authors:** Anna Burguin, Caroline Diorio, Francine Durocher

**Affiliations:** 1 Department of Molecular Medicine, Faculty of Medicine, Université Laval, Quebec City, QC G1T 1C2, Canada; anna.burguin@crchudequebec.ulaval.ca; 2 Cancer Research Center, CHU de Québec-Université Laval, Quebec City, QC G1V 4G2, Canada; caroline.diorio@crchudequebec.ulaval.ca; 3 Department of Preventive and Social Medicine, Faculty of Medicine, Université Laval, Quebec City, QC G1T 1C2, Canada

**Keywords:** breast cancer, personalized therapies, molecular subtypes, breast cancer treatment, luminal, HER2, TNBC

## Abstract

Breast cancer (BC) is the most frequent cancer diagnosed in women worldwide. This heterogeneous disease can be classified into four molecular subtypes (luminal A, luminal B, HER2 and triple-negative breast cancer (TNBC)) according to the expression of the estrogen receptor (ER) and the progesterone receptor (PR), and the overexpression of the human epidermal growth factor receptor 2 (HER2). Current BC treatments target these receptors (endocrine and anti-HER2 therapies) as a personalized treatment. Along with chemotherapy and radiotherapy, these therapies can have severe adverse effects and patients can develop resistance to these agents. Moreover, TNBC do not have standardized treatments. Hence, a deeper understanding of the development of new treatments that are more specific and effective in treating each BC subgroup is key. New approaches have recently emerged such as immunotherapy, conjugated antibodies, and targeting other metabolic pathways. This review summarizes current BC treatments and explores the new treatment strategies from a personalized therapy perspective and the resulting challenges.

## 1. Introduction

Breast cancer (BC) is the most frequent cancer and the second cause of death by cancer in women worldwide. According to Cancer Statistics 2020, BC represents 30% of female cancers with 276,480 estimated new cases and more than 42,000 estimated deaths in 2020 [1]. 

**Figure 1:**
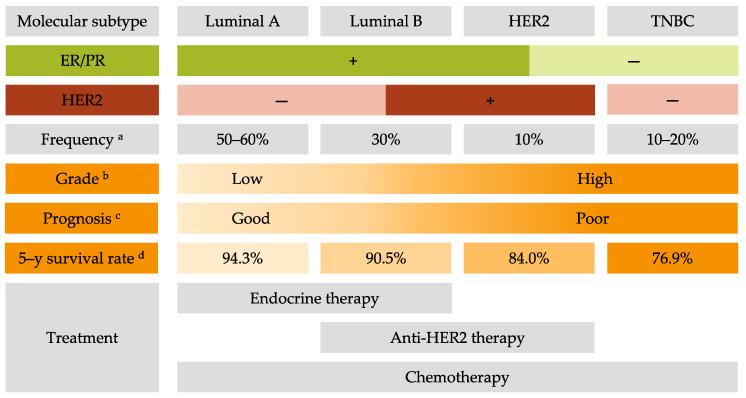
Characteristics of breast cancer molecular subtypes. ER: estrogen receptor; PR: progesterone receptor; HER2: human epidermal growth factor receptor 2; TNBC: triple-negative breast cancer. ^a^. Frequency derived from Al-thoubaity et al. [12] and Hergueta-Redondo et al. [13]. ^b^. Grade derived from Engstrom et al. [14]. ^c^. Prognosis derived from Hennigs et al. [15] and Fragomeni et al. [16]. ^d^. The 5–year survival rate derived from the latest survival statistics of SEER [7].

Invasive BC can be divided into four principal molecular subtypes by immunohistological technique based on the expression of the estrogen receptor (ER), the progesterone receptor (PR), and the human epidermal growth factor receptor 2 (HER2) [2]. Luminal A BC (ER+ and/or PR+, and HER2-) represents around 60% of BC and is associated with a good prognosis [3]. Luminal B BC (ER+ and/or PR+, and HER2+) represents 30% of BC and is associated with high ki67 (>14%), a proliferation marker, and a poor prognosis [4]. HER2 BC (ER-, PR-, and HER2+) represents 10% of BC and is also associated with a poor prognosis [5]. Lastly, triple-negative BC (TNBC) (ER-, PR-, and HER2-) represents 15–20% of BC and is associated with more aggressivity and worse prognosis compared to other BC molecular subtypes and often occurs in younger women [6]. Characteristics of BC by molecular subtypes are described in Figure 1. 

The 5-year relative BC-specific survival rate of BC is encouraging with 90.3% for all subtypes and stages. However, for metastatic BC the 5-year relative cancer-specific survival rate is still low: 29% regardless of subtype and can drop to 12% for metastatic TNBC [7]. This clearly indicates that strategies of treatment for metastatic BC patients are not effective enough to ensure a good survival rate. Thus, it is crucial to find new solutions for the treatment of metastatic BC and especially TNBC. 

Treatment choice is based on the grade, stage, and BC molecular subtype to have the most personalized, safe, and efficient therapy. The grade describes the appearance of tumor cells compared to normal cells. It includes tubule differentiation, nuclear pleomorphism, and the mitotic count [8]. The stage is used to classify the extent of cancer in the body and is defined using the TNM system comprising tumor size, lymph node status, and the presence of metastases [9]. For non-metastatic BC, the strategic therapy involves removing the tumor by complete or breast-conserving surgery with preoperative (neoadjuvant) or postoperative (adjuvant) radiotherapy and systemic therapy including chemotherapy, and targeted therapy. Targeted therapy comprises endocrine therapy for hormone receptor-positive (HR+) BC and anti-HER2 therapy for HER2+ BC. Unfortunately, there is no available targeted therapy for the TNBC subtype. For metastatic BC the priority is to contain tumor spread as this type of BC remains incurable. The same systemic therapies are used to treat metastatic BC [10]. 

Challenges in the treatment of BC including dealing with treatment resistance and recurrence. Indeed, 30% of early-stage BC have recurrent disease, mostly metastases [11]. Thus, it is crucial to develop new strategic therapies to treat each BC subgroup effectively. 

This review will summarize current treatments for invasive BC, the underlying resistance mechanisms and explore new treatment strategies focusing on personalized therapy and the resulting challenges.

## 2. Common Treatments for All Breast Cancer Subtypes 

In addition to surgery, radiotherapy and chemotherapy are used routinely to treat all BC subtypes [17].

### 2.1. Surgery

The most standard breast surgery approaches are either total excision of the breast (mastectomy), usually followed by breast reconstruction, or breast-conserving surgery (lumpectomy). Lumpectomy entails the excision of the breast tumor with a margin of surrounding normal tissue. The recommended margins status is defined as “no ink on tumor”, meaning no remaining tumor cells at the tissue edge [18]. Studies show that total mastectomy and lumpectomy plus irradiation are equivalent regarding relapse-free and overall survival (OS) [19]. Contraindications for breast-conserving surgery include the presence of diffuse microcalcifications (suspicious or malignant-appearing), disease that cannot be incorporated by local excision with satisfactory cosmetic result, and *ATM* (ataxia-telangiesctasia mutated) mutation (biallelic inactivation) [18]. 

The surgery to remove axillary lymph nodes is useful to determine cancerous cell spread and for therapeutic purposes. For instance, axillary lymph node dissection (ALND) can improve survival rated by removing remaining tumor cells. ALND used to be the goal standard for removing positive lymph nodes. However, clinical trials showed that sentinel lymph node biopsy (SLNB) had the same effect as ALND regarding disease-free survival (DFS) and OS [20]. Other clinical trials demonstrated that ALND was not necessary for all patients with positive lymph nodes. Moreover, most patients who receive radiation and systemic treatment after SLNB have negative lymph nodes as these treatments are sufficient in eliminating residual tumor cells [21]. 

### 2.2. Radiotherapy 

Radiation therapy has been used to treat cancer since Röngten discovered the X-ray in 1895 [22]. High-energy radiations are applied to the whole breast or a portion of the breast (after breast-conservative surgery), chest wall (after mastectomy), and regional lymph nodes [23]. A meta-analysis showed that radiation following conservative surgery offered more benefits to patients with higher-risk BC while patients with small, low-grade tumors could forego radiation therapy [24]. Postmastectomy radiation to the chest wall in patients with positive lymph nodes is associated with decreased recurrence risk and BC mortality compared to patients with negative lymph nodes [25]. A radiation boost to the regional node radiation treatment can be incorporated after mastectomy for patients at higher risk for recurrence [26]. This additional radiation boost to regional nodes following mastectomy is associated with improved (DFS) but is also associated with an increase in radiation toxicities such as pneumonitis and lymphedema [27]. Radiotherapy can be administered concurrently with personalized therapy (anti-HER2 therapy or endocrine therapy). 

As one of the major side effects of radiotherapy is cardiotoxicity, it is critical to minimize exposure to the heart and lungs [28]. Additional techniques can be used to reduce the radiation exposure to the heart, lungs, and normal tissue such as prone positioning, respiratory control, or intensity-modulated radiotherapy [29]. 

Advanced invasive BC can exhibit radiation therapy resistance [30]. The hypoxic tumor microenvironment, which lacks oxygen, leads to increased cell proliferation, apoptosis resistance, and radiotherapy resistance [31]. The major player of this resistance is the HIF-1α (hypoxia-inducible factor 1 alpha) protein [32]. Indeed, HIF-1α overexpression is caused by low oxygen levels within the microenvironment and promotes the maintenance of hypoxia by allowing tumoral cells to survive in a hypoxic microenvironment [33,34,35]. Cancer stem cells (CSC) could also have a role in radiation therapy resistance [36]. CSC can self-renew and initiate subpopulations of differential progeny, and a hypoxic microenvironment is ideal for CSC survival and proliferation [37,38]. 

Radiation therapy is used to treat all BC subtypes, but its implication is more important for TNBC, as there is no personalized therapy for this subtype. It has been shown that radiotherapy benefits TNBC patients both after conserving surgery and mastectomy [39]. 

### 2.3. Chemotherapy

BC chemotherapy comprises several families of cytotoxic drugs, including alkylating agents, antimetabolites and tubulin inhibitors [40]. Cyclophosphamide is a nitrogen mustard alkylating agent causing breakage of the DNA strands [41]. The mechanism of action for anthracyclines (doxorubicin, daunorubicin, epirubicin, and idarubicin) includes DNA intercalation, thereby inhibiting macromolecular biosynthesis [42]. Taxanes, including docetaxel and paclitaxel, bind to microtubules and prevent their disassembly, leading to cell cycle arrest and apoptosis [43]. 

Chemotherapy can be administered in the neoadjuvant or adjuvant setting and for metastatic BC treatment. 

#### 2.3.1. Neoadjuvant Chemotherapy (NAC)

Neoadjuvant chemotherapy was initially administered for non-metastatic but inoperable BC, defined as unreachable tumors [44]. Then, chemotherapy was used before the surgery for operable tumors to facilitate breast conservation [45]. 

Studies demonstrated that chemotherapy administered before surgery is as effective as administered after surgery [46,47,48]. The NSABP-B-18 trial compared the effects of doxorubicin and cyclophosphamide administered either postoperatively or preoperatively. This trial showed that NAC reduces the rate of axillary metastases in node-negative BC patients [48]. 

Some patients fail to achieve pathologic complete response after a full course of NAC. Unfortunately, there is no consensus regarding the treatment strategy to follow for patients with residual disease after surgery [49,50]. The BC subtype plays an important role in the response to NAC. Indeed, TNBC and HER2+ BC are more likely to be sensitive to chemotherapy. Hence, NAC is a good strategy to maximize pathologic complete response in these BC subtypes [45]. 

#### 2.3.2. Adjuvant Chemotherapy 

Adjuvant chemotherapy is administered to BC patients with lymph nodes metastases or a high risk of recurrence [51]. The standard chemotherapy treatment comprises an anthracycline and a taxane. The two most common regimens are cyclophosphamide and doxorubicin for four cycles followed by paclitaxel for four cycles. Then patients are given the previous combination of therapies followed by either weekly paclitaxel for 12 weeks, or docetaxel every 3 weeks for four cycles [52,53]. 

Like neoadjuvant therapy, patients with HR-negative BC receive more benefits from adjuvant therapy (i.e., reduction of BC recurrence and mortality) than HR+ BC patients [54]. However, for patients with HR+, node-negative BC associated with a high Oncotype recurrence score (≥31), calculated from the expression of 16 BC-related genes and 5 reference genes, adjuvant chemotherapy reduces the risk of recurrence [55]. The TAILORx clinical trial showed that HR+ BC patients with a low Oncotype recurrence score do not benefit from chemotherapy alone [56]. 

According to the molecular BC subtype, chemotherapy can be administered with targeted therapies. Patients with HR+ BC should receive endocrine therapy after chemotherapy is completed, and HER2+ BC patients should receive trastuzumab combined with chemotherapy [57]. For TNBC patients, front-line therapy includes a combination of taxane and anthracycline [58]. 

One of the major drawbacks of chemotherapy is its side effects. The early side effects (0–6 months of treatment) involve fatigue, alopecia, cytopenia (reduction in the number of normal blood cells), muscle pain, neurocognitive dysfunction, and chemo-induced peripheral neuropathy. The chronic or late side effects (after 6 months of treatment) include cardiomyopathy, second cancers, early menopause, sterility, and psychosocial impacts [59]. 

As mentioned previously in this review, chemotherapy is composed of taxanes, anthracyclines and cyclophosphamide. Each of these molecules can lead to resistance in BC patients [60]. 

One mechanism of resistance is by overexpressing p-glycoprotein, an ATP-binding cassette (ABC) family member, which confers resistance to anthracycline and taxanes [61]. Breast cancer resistance protein (BCRP), another ABC family member, induces resistance to anthracycline but not taxanes when overexpressed [62]. Microtubule alterations can also lead to taxane resistance. The overexpression of β-tubulin III induces paclitaxel resistance [63]. Moreover, mutations in microtubule-associated proteins (MAPs) affect microtubule dynamics and improve taxane resistance [64]. Multiple enzymes are known to be involved in the cyclophosphamide detoxification, leading to its resistance. For example, aldehyde dehydrogenase upregulation detoxifies aldophosphamide a type of cyclophosphamide, and mutations in glutathione S-transferases, enzymes involved in drug-metabolizing conjugation reactions, can also affect cyclophosphamide detoxification [65,66]. 

Surgery, radiotherapy, and chemotherapy are complementary strategies in the treatment of BC patients. However, they are not sufficient to effectively treat all BC molecular subtypes, as they do not have the same response to radiotherapy or chemotherapy. Thus, personalized therapies are essential in the process for BC treatment. 

## 3. Current Personalized Treatments for Breast Cancer: Strengths and Weaknesses 

**Figure 2:**
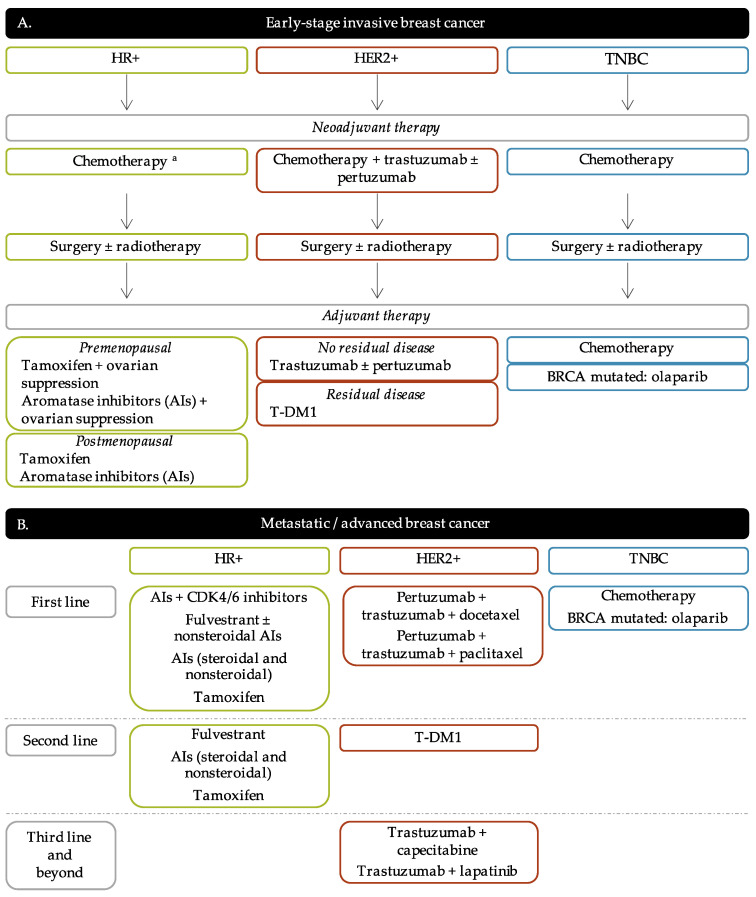
Breast cancer treatment flow diagram. (**A**). Early-stage breast cancer. (**B**). Metastatic/advanced breast cancer. ^a^ Neoadjuvant chemotherapy for HR+ BC patients is not systematic. It is mainly administered to luminal B BC patients and/or elder BC patients. HR+: hormone receptors positive; HER2+: human epidermal growth factor receptor 2 positive; TNBC: triple-negative breast cancer; AIs: aromatase inhibitors; T-DM1: trastuzumab-emtansine.

The current strategies of treatment are principally based on the tumor progression and BC molecular subtypes in order to offer the most personalized treatment for BC patients. The algorithm of BC treatment is represented in Figure 2. 

### 3.1. Endocrine Therapy

**Figure 3:**
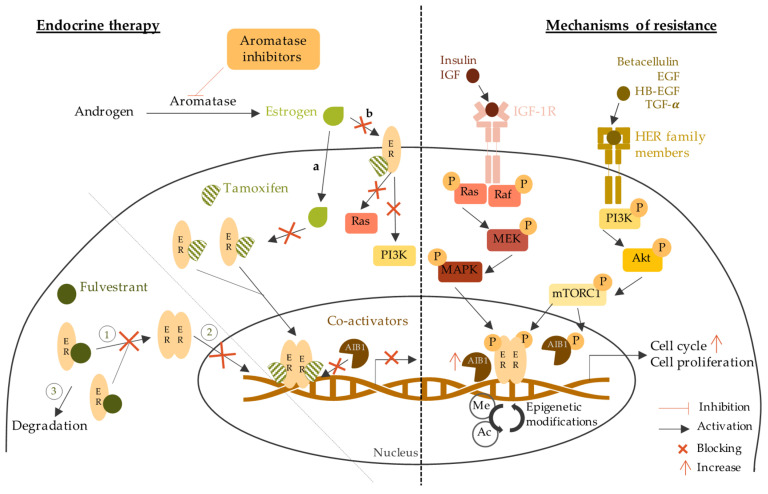
Endocrine therapy mechanisms of action and resistance. The left part of the figure shows the mechanism of endocrine therapy through aromatase inhibitors, tamoxifen, and fulvestrant. The right part of the figure describes the mechanisms of resistance to endocrine therapy through the epigenetic modifications, the increase of coactivators and cell cycle actors, and the activation of other signaling pathways. Estrogens can go through the plasma membrane by a. diffusion as they are small non-polar lipid soluble molecules; b. binding to membrane ER initiating the activation of Ras/Raf/MAPK and PI3K/Akt signaling pathways which are blocked by tamoxifen. 1: inhibition of ER dimerization; 2: blockage of nucleus access; 3: ER degradation. ER: estrogen receptor; AIB1: amplified in breast cancer 1; IGF-1R: insulin growth factor receptor 1; IGF: insulin growth factor; HER: human epidermal receptors; EGF: epidermal growth factor; HB-EGF: heparin-binding EGF-like growth factor; TGF-α: transforming growth factor alpha; MEK/MAPK: mitogen activated protein kinase; PI3K: phosphoinositide 3-kinase; mTOR: mammalian target of rapamycin; Me: methylation; Ac: acetylation.

Endocrine therapy is the main strategy to treat HR positive invasive BC. The purpose of this therapy is to target the ER directly (selective estrogen receptors modulators and degraders) or the estrogen synthesis (aromatase inhibitors) [67]. The most common types of endocrine therapy are selective estrogen receptor modulators (SERMs), selective modulators estrogen receptor degraders (SERDs), and aromatase inhibitors (AIs) [68]. Endocrine therapy mechanism of action and resistance are described in Figure 3.

#### 3.1.1. Selective Estrogen Receptor Modulators (SERMs)

SERMs, such as tamoxifen, toremifene, bazedoxifene, and raloxifene, are antiestrogens that compete with estrogen by binding to the ER. This binding changes the conformation of the ER ligand-binding domain, and once ER is translocated to the nucleus, it blocks co-factor recruitment and subsequent genes transcription involved in cell cycle progression (cyclin D1), cell proliferation (like IGF-1), or cell migration (collagenase) [69,70]. 

The most used SERMs is tamoxifen, approved by the US Food and Drugs Administration (FDA) in 1977. It is an adjuvant therapy orally administered for 5 to 10 years according to tumor aggressivity. Tamoxifen adjuvant treatment reduces recurrence risk by 50% for the first 5 years and 30% for the next 5 years [71]. Tamoxifen is given to either premenopausal or postmenopausal patients. However, for high-risk premenopausal patients, adding ovarian suppression is more effective than tamoxifen alone [72]. Tamoxifen can also be administered as neoadjuvant treatment, especially for elderly BC patients [73]. However, studies have demonstrated no difference in OS for ER+ BC patients when neoadjuvant tamoxifen is compared to surgery [74,75].

Other SERMs have since been developed, such as toremifene approved by the FDA in 1997 [76]. Studies comparing the effect of toremifene and tamoxifen in premenopausal patients with ER+ advanced BC have shown that toremifene efficacy and safety are similar to tamoxifen [77,78]. Bazedoxifene and raloxifene are administered as prevention treatment to postmenopausal patients at high risk of developing invasive BC and for preventing osteoporosis [79,80,81]. 

The most frequent adverse events of SERMs are hot flushes, nausea, vomiting, vaginal bleeding/discharges, and increased risk of thromboembolic events [82]. Of note, about 40% of HR+ BC patients will develop resistance to SERMs [83]. SERMs resistance can occur by the loss of ER expression or functions. Epigenetic modifications such as hypermethylation of CpG islands or histone deacetylation can lead to transcriptional repression of ER [84]. Another potential mechanism for ER expression loss is the overpopulation of ER-negative cells in heterogenous ER+ tumors [85]. Mutations in the ligand-binding domain of ER gene (*ESR1*) inhibit the binding of estrogen to the ER leading to the abolition of downstream signaling. Moreover, abnormal splicing can lead to truncated, nonfunctional ER protein [86,87]. Another explanation for SERMs resistance is the abnormal expression of ER coregulators [88]. Coregulators are very important in the ER pathway as they can increase or decrease ER activity depending on incoming signals [89]. The most studied coregulator involved in SERMs resistance is the AIB1 (Amplified in breast cancer 1) coactivator protein, often overexpressed in resistant breast tumors [90]. In particular, in ER+ cells that overexpress HER2, there is a crosstalk between HER2 and AIB1. HER2 induces phosphorylation of AIB1 leading to evasion and subsequent activation of the ER signaling pathway even though it is inhibited by SERMs [91] 

#### 3.1.2. Selective Estrogen Receptor Degraders (SERDs)

To counteract the large proportion of tamoxifen-resistant tumors, a new type of therapeutic agents with a different mechanism of action has been developed: SERDs. In contrast to SERMs, SERDs completely block the ER signaling pathway. 

Fulvestrant is the main SERD administered. It was discovered by Wakeling and collaborators in 1987 and demonstrated pure anti-estrogen activity [92]. Fulvestrant binds to ER with a higher affinity than tamoxifen. Once it binds to the ER, it inhibits receptor dimerization and then blocks ER translocation to the nucleus leading to its degradation [93,94,95].

Fulvestrant is administered by intramuscular injections, and common adverse effects are nausea, pain, and headaches [96]. Fulvestrant is approved to treat postmenopausal and premenopausal patients with ovarian function suppression, with ER+ advanced or metastatic BC on prior endocrine therapy [97]. More recently (in 2017), fulvestrant was approved as first-line monotherapy for advanced ER+ breast cancer [98]. According to the 2021 NCCN guidelines, fulvestrant combined with endocrine therapy or CDK4/6 inhibitors is one of the preferred regimens for second-line therapy in ER+ advanced or metastatic BC [99]. The combination of fulvestrant with other endocrine therapies has not shown any advantages over fulvestrant used in monotherapy [100,101]. Clinical studies have shown benefits from fulvestrant when administered in higher doses to patients with *ESR1*-mutated advanced BC [102,103]. Indeed, *ESR1* mutations occur in nearly 20% of cases of ER+ BC [86].

However, fulvestrant can lead to resistance by different mechanisms. For example, by upregulating the PI3K (phosphatidylinositol 3-kinase), mTOR (mammalian target of rapamycin) and Ras-ERK (extracellular signal-regulated kinase) signaling pathways. PI3K/Akt/mTOR is a downstream signaling pathway of ER activation and plays an important role in antiestrogen therapy resistance [104]. PI3K pathway activation can occur independently of ER by binding to the epidermal growth factor (EGF) [105]. Moreover, it has been shown that Akt overexpression leads to fulvestrant resistance [106]. IGF-1R activation (insulin-like growth factor 1 receptor) may be another mechanism of resistance to fulvestrant. IGF-1R expression is involved in cell survival and promotes metastatic cell proliferation. The interaction between IGF-1R and ER initiates the activation of IGF-1R/MAPK (mitogen-activated protein kinase) and IGF-1R/PI3K signaling leading to antiestrogen resistance [107].

#### 3.1.3. Aromatase Inhibitors (AIs)

Aromatase is a cytochrome P50 enzyme involved in the synthesis of androgens and estrogens [108]. Aromatase is found in the breast, uterus, and other estrogen-sensitive tissues in specific levels depending on menopausal status [109,110]. Aromatase expression is increased in breast tumors and associated with high estrogen levels. Therefore, high expression of aromatase promotes ER+ tumor proliferation [111]. 

Aromatase inhibitors (AIs) block aromatase enzyme activity, leading to the inhibition of estrogen synthesis. Current AIs can be classified into two categories: steroidal AIs and non-steroidal AIs [112]. Exemestane, a steroidal AI, has a steroid-like structure similar to androstenedione, which is the aromatase substrate. Exemestane irreversibly binds to the aromatase substrate-binding site leading to its inactivation [113]. Non-steroidal AIs include letrozole and anastrozole. They both bind non-covalently and competitively to the aromatase substrate-binding site and prevent the binding of androgens by saturating the binding site [112]. 

AIs are an oral treatment administered only to postmenopausal women (including patients that become postmenopausal following ovarian suppression). It is administered alone or in combination with tamoxifen as adjuvant therapy for HR+ BC patients [114,115,116,117]. AIs can be administered for 5 years or 2–3 years if followed by tamoxifen and up to 5 years after previous tamoxifen or AI treatment. For advanced or metastatic HR+ BC, AIs can be delivered as first-line and second-line therapy. Patients who become postmenopausal after or during the 5 years of tamoxifen treatment can receive AIs, such as letrozole, as an extended treatment strategy [118,119]. 

Estrogens have protective effects on the cardiovascular system by regulating serum lipids concentrations and increasing vasodilatation [120]. Hence, AIs might increase the risk of developing cardiovascular diseases by reducing estrogen levels in the blood [121]. Other adverse effects of AIs include hot flushes, vaginal dryness, fatigue, and osteoporosis [122]. ER+ tumors can acquire AI resistance. Some mechanisms of AI resistance are similar to those conferring SERM or SERD resistance, such as *ESR1* mutations, epigenetic modifications, and PI3K pathway upregulation [123]. However, other mechanisms of action are involved in AI resistance. For example, the upregulation of cyclin-dependent kinase 4 (CDK4) or cyclin-dependent kinase 6-retinoblastoma (CDK6-RB) pathways can lead to an estrogen-dependent cell progression [124]. Clinical studies have shown better benefits from CDK4-CDK6 inhibitors in combination with AIs compared to AIs alone [125,126]. 

Endocrine therapy is a well-established treatment strategy for HR+ tumors. Over the last decades, SERMs, SERDs and AIs have been proven as safe and effective personalized therapy for HR+ BC patients, and these therapeutic strategies have shown continued improvements. However, the main drawback of endocrine therapy is acquired or *de novo* resistance [127]. Hence, it is essential to develop new therapeutic agents that use different modes of action to treat HR+ BC more efficiently.

### 3.2. Anti-HER2 Therapy 

**Figure 4:**
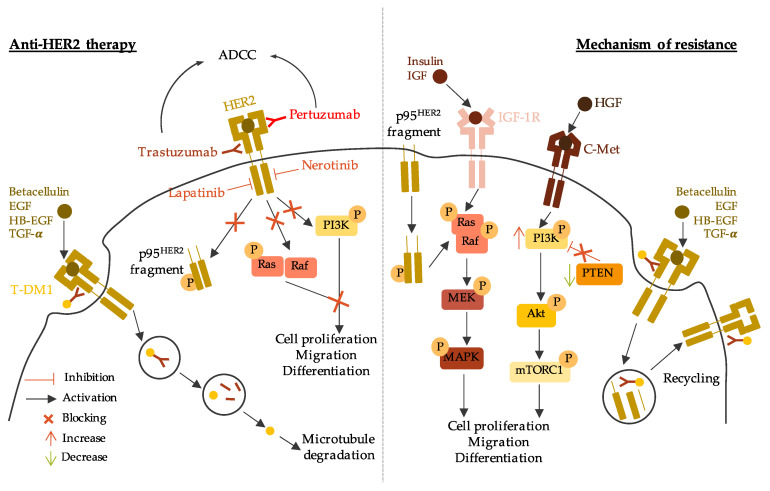
Anti-HER2 therapy mechanisms of action and resistance. The left part of the figure describes the mechanism of action of anti-HER2 therapy through anti-HER2 antibody (trastuzumab and pertuzumab), tyrosine kinase inhibitors (lapatinib and nerotinib), and trastuzumab-emtansine (T-DM1). The right part of the figure describes the mechanism of resistance to anti-HER2 therapy through constitutive active p95^HER2^ fragment, activation of other signaling pathways, and rapid recycling of HER2-T-DM1. ADCC: antibody-dependent cellular cytotoxicity; HER2: human epidermal growth factor receptor 2; EGF: epidermal growth factor, HB-EGF: heparin-binding EGF-like growth factor; TGF-α: transforming growth factor alpha; T-DM1: trastuzumab-emtansine; IGF-1R: insulin growth factor receptor 1; IGF: insulin growth factor; HGF: hepatocyte growth factor; MEK/MAPK: mitogen activated protein kinase; PI3K: phosphoinositide 3-kinase; mTOR: mammalian target of rapamycin; PTEN: phosphatase and tensin homolog.

The overexpression of HER2 is associated with worse survival outcome compared to HR-positive/HER2-negative BC [128,129]. Hence, therapies targeting HER2 are essential to treat HER2-positive BC. The current anti-HER2 therapies comprise antibodies that target specific HER2 epitopes, tyrosine kinase inhibitors (TKIs) and, more recently, antibody-drug conjugates (ADCs) [130]. Anti-HER2 mechanisms of action and resistance are described in Figure 4. 

#### 3.2.1. Antibodies Targeting HER2

The first developed HER2-targeted antibody, trastuzumab (Herceptin), was approved by the FDA in 1998 [131,132]. Trastuzumab targets subdomain IV of the HER2 extracellular domain. However, the mechanism underlying trastuzumab’s therapeutic effect is not well understood. Multiple studies have reported hypotheses to explain trastuzumab’s mechanism of action. For instance, trastuzumab may inhibit the formation of the HER2-HER3 heterodimer, known to be the most oncogenic pair in the HER family [133]. It could also inhibit the formation of the active p95^HER2^ fragment by preventing cleavage of the HER2 extracellular domain [134]. An indirect antitumor effect could be activating antibody-dependent cellular cytotoxicity (ADCC) by engaging with Fc receptors on immune effector cells [135]. 

Initially, trastuzumab was approved for administration in metastatic HER2+ BC, increasing the clinical benefits of first-line chemotherapy [132]. Trastuzumab has also demonstrated its efficacy and safety in early-stage HER2+ BC. It is given as neoadjuvant or adjuvant therapy in combination with other anti-HER2 treatments and/or with chemotherapy [136,137,138]. The recommended dose for intravenous trastuzumab is 4 mg/kg followed by 2 mg/kg weekly for 1 year in the adjuvant setting for early-stage HER2+ BC and until disease-free progression for metastatic HER2+ BC [139]. 

Pertuzumab (Perjeta) is another antibody that targets the HER2 extracellular domain but binds to subdomain II. Once it binds to HER2, pertuzumab prevents HER2 heterodimerization with other HER family members, leading to inhibition of downstream signaling pathways [140]. Like trastuzumab, one of pertuzumab’s indirect antitumor effects is activating the ADCC pathway [141]. Multiple clinical trials have shown that pertuzumab, combined with trastuzumab and chemotherapy, improved OS in metastatic HER2+ BC patients compared to trastuzumab and chemotherapy alone [142,143,144,145]. The benefits of pertuzumab have also been shown in early-stage HER2+ BC, as pertuzumab can be used in the neoadjuvant or adjuvant setting combined with trastuzumab and chemotherapy [146,147,148,149]. Pertuzumab is administered in fixed doses of 840 mg followed by 420 mg every three weeks [150]. 

Despite the major positive impacts of trastuzumab and pertuzumab in HER2+ BC treatment, only one-third of BC patients with HER2+ tumors benefit from anti-HER2 antibodies [151]. One of the hypotheses explaining this resistance concerns structural modifications of HER2, which hinder antibody binding. Alternative splicing can lead to a truncated isoform lacking the extracellular domain, thus forming a constitutive active p95^HER2^ fragment [152]. The overexpression of other tyrosine kinases can bypass the signaling pathways mediated by HER2. It has been shown that cells overexpressing IGF-1R overcome cell cycle arrest by increasing CDK2 kinase activity [153]. Moreover, the overexpression of c-Met (a hepatic growth factor receptor) synergizes with HER2 signaling to confer resistance to anti-HER2 antibodies. Indeed, c-Met physically interacts with HER2, and c-Met depletion renders cells more sensitive to trastuzumab [154,155]. Another hypothesis for anti-HER2 antibody resistance is intracellular alterations in HER2 downstream signaling pathways. HER2 activates PI3K/Akt signaling, and PTEN (phosphatase and tensin homolog) is a well-known inhibitor of this pathway [156]. Tumors with a loss of PTEN function and/or constitutive activation of PI3K due to alteration mutations achieve worse therapeutic outcomes with trastuzumab [157,158].

#### 3.2.2. Tyrosine Kinase Inhibitors (TKIs)

Since tumors may be resistant to anti-HER2 antibodies, new approaches have been developed. TKIs such as lapatinib, neratinib, or pyrotinib are small molecules that compete with ATP at the catalytic domain of the receptor to prevent tyrosine phosphorylation and HER2 downstream signaling [159]. 

Lapatinib is a dual EGFR/HER2 TKI blocking both HER1 and HER2 activation [160]. In metastatic BC, clinical trials have shown that lapatinib offers more benefits than chemotherapy alone [161,162,163]. The effects of lapatinib in the neoadjuvant/adjuvant setting have also been evaluated. As a neoadjuvant treatment, lapatinib plus trastuzumab combined with chemotherapy were more efficient than chemotherapy combined with lapatinib or trastuzumab alone [164]. Lapatinib as adjuvant treatment showed modest antitumor efficacy compared to placebo in a randomized, controlled, and multicenter phase III trial (TEACH) [165]. For luminal B (ER/PR+; HER2+) advanced or metastatic BC, lapatinib can be administered in combination with AIs. 

Neratinib is an irreversible TKI targeting HER1, HER2, and HER4 [166]. The FDA approved Neratinib in 2017 as an extended adjuvant treatment for patients with HER2+ early-stage BC and combination with trastuzumab in the adjuvant setting [167,168]. Neratinib can be delivered in combination with capecitabine as a third-line and beyond therapy for HER2+ advanced or metastatic BC. 

More recently, pyrotinib, a new generation TKI targeting HER1, HER2 and HER4, has been developed [169]. Pyrotinib is still under clinical trials to prove its efficacy and safety [170]. However, in 2018, the Chinese State Drug Administration approved pyrotinib in combination with or after chemotherapy treatment for patients with HER2+ advanced or metastatic BC [171]. 

Despite the recent development of TKI treatments, patients can still exhibit intrinsic or acquired resistance to these agents. Three mechanisms of action have been hypothesized: (1) activation of compensatory pathways, (2) HER2 tyrosine kinase domain mutation, and (3) other gene amplification [172]. For instance, activation of the PI3K/Akt pathway and FOXO3A (Forkhead transcription factor) by the upregulation of HER3 can lead to lapatinib resistance [173]. Other tyrosine kinases can be involved, such as c-Met, also known to be implicated in trastuzumab resistance. C-Met induces the activation of PI3K/Akt signaling in lapatinib-resistant BC [174]. Mutations in the HER2 tyrosine kinase domain lead to the constitutive activation of HER2 by substituting individual amino acids [175]. Lastly, it has been shown that the amplification of the *NIBP* (TRAPPC9, Trafficking Protein Particle Complex 9) gene occurs in HER2+ lapatinib-resistant tumors. The inhibition of NIBP makes resistant cells sensitive to lapatinib [176]. 

#### 3.2.3. Trastuzumab-Emtansine (T-DM1)

Trastuzumab-emtansine (T-DM1) is an antibody-drug conjugate (ADC), which is a conjugate of trastuzumab and a cytotoxic molecule, DM1, a derivative of maytansine [177]. T-DM1 binds to HER2 with the trastuzumab part. The formed complex is then internalized for degradation, releasing DM1 metabolites into the cytoplasm. DM1 then inhibits microtubule assembly causing cell death [178,179]. Thus, T-DM1 consists of the antitumor effects of trastuzumab and those associated with DM1 metabolites [180]. 

Three phase III clinical trials have evaluated the safety and efficacy of T-DM1 for HER2+ metastatic BC [181,182,183]. They have shown that T-DM1 improves OS and DFS of HER2+ metastatic BC patients compared to lapatinib in combination with trastuzumab or chemotherapy [181,182,183]. T-DM1 as neoadjuvant treatment has less efficacy compared with trastuzumab or pertuzumab with chemotherapy [146]. This suggests that T-DM1 should not be administered as a neoadjuvant treatment but as a first-line or second-line therapy for HER2+ metastatic BC. The 2021 NCCN guidelines recommend using T-DM1 as second-line therapy for HER2+ advanced or metastatic BC [99]. 

The mechanism of action of T-DM1 involves those related to trastuzumab and DM1, so the observed resistance to T-DM1 could come from interference in one or both constituents [184]. The mechanism of T-DM1 resistance has been hypothesized to involve (1) the loss of trastuzumab mediated activity, (2) the dysfunctional intracellular trafficking of T-DM1, and (3) the impairment of DM1 mediated cytotoxicity [185]. 

As previously described in this review, the reduction of trastuzumab effects can occur by reduced HER2 expression, dysregulation of PI3K signaling, or the activation of alternative tyrosine kinase receptors [153,154,156,186]. The alteration of HER2-T-DM1 complex internalization can go through a rapid recycling of HER2 to the plasma membrane leading to the inhibition of DM1 metabolism released into the cytoplasm [187]. The internalization of the HER2-T-DM1 complex occurs through the formation of lysosomes. These vesicles enclose lysosomal enzymes involved in HER2-T-DM1 complex degradation. In T-DM1-resistant tumors, the level of lysosomal enzymes is inhibited [188,189]. T-DM1 also disrupts microtubule assembly causing incomplete spindle formation resulting in mitotic catastrophe and apoptosis [190]. Cells resistant to T-DM1 can avoid this process by reducing the induction of Cyclin-B1, an enzyme essential for cell cycle progression [191].

HER2+ BC are aggressive and associated with poor prognosis and metastasis, and recurrences. Anti-HER2 therapy has greatly improved the management of HER2+ BC. However, 25% of early-stage HER2+ BC patients will have a recurrence after the initial anti-HER2 treatment [192]. The emergence of new therapeutic agents specific for HER2+ BC provides new hope to treat this particularly aggressive BC subtype.

### 3.3. PARP Inhibitors 

The prevalence of *BRCA* (Breast Cancer genes) mutations in TNBC patients is approximately 20% [193]. BRCA1 and BRCA2 are proteins involved in the DNA damage response to repair DNA lesions [194]. Mutations in *BRCA 1/2* genes are associated with an increased risk of breast and ovarian cancers [195]. 

**Figure 5:**
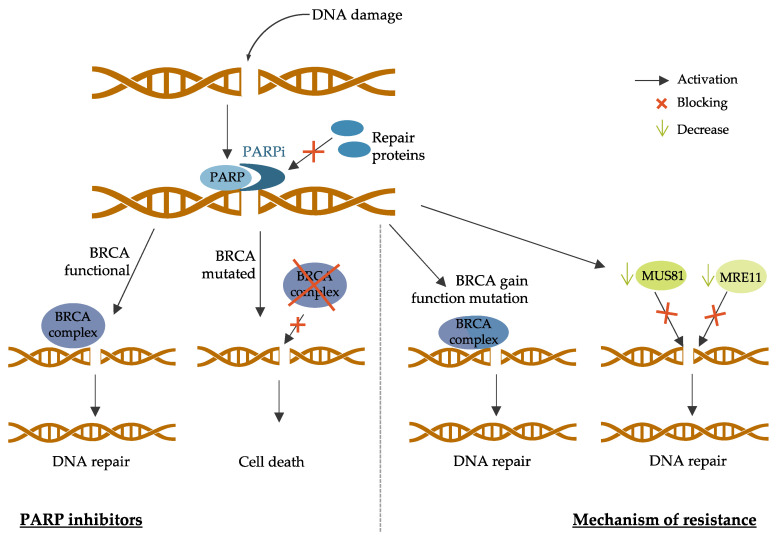
PARP inhibitors mechanisms of action and resistance. The left part of the figure describes the mechanism of PARP inhibitors in the context of BRCA mutated breast cancer. The right part of the figure describes the mechanism of resistance to PARP inhibitors through secondary intragenic mutations restoring BRCA proteins functions and the decrease of the recruitment of nucleases (MUS81 or MRE11) to protect the replication fork. PARP: poly-(ADP-ribose) polymerase protein; PARPi: PARP inhibitors; BRCA: breast cancer protein; MUS81: methyl methanesulfonate ultraviolet sensitive gene clone 81; MRE11: meiotic recombination 11.

PARP (poly-(ADP-ribose) polymerase protein) proteins are also involved in the DNA damage response as they recruit DNA repair proteins, such as BRCA1 and BRCA2, to the damage site [196]. PARP inhibitors (PARPi) were developed to inhibit DNA repair in BRCA-mutated BC since cells defective in BRCA functions cannot repair DNA damage when PARP is inhibited [197]. The principal PARPis currently in clinical development are olaparib, talazoparib, veliparib, and rucaparib [198]. PARP inhibitors mechanisms of action and resistance are described in Figure 5. 

#### 3.3.1. Olaparib 

Olaparib is the first FDA-approved PARPi for the treatment of *BRCA*-mutated BC [199]. Phase I and phase II trials evaluating the effects of olaparib monotherapy in germline BRCA-mutated (gBRCAm) BC proved its clinical benefits by improving progression-free survival (PFS) [200,201,202,203]. The phase III, randomized, open-label, OlympiAD trial compared olaparib monotherapy vs. standard chemotherapy in patients with BRCA mutated HER2-negative BC. This trial showed that olaparib has better efficacy and tolerability than standard chemotherapy for this group of patients [204]. Olaparib has also been tested in combination with chemotherapy. A phase I study evaluated the effects of olaparib in combination with paclitaxel in unselected TNBC patients [205]. The overall response rate (ORR) for these patients was 37%. Two phase I studies evaluating the combination of olaparib with cisplatin or carboplatin in gBRCAm BC patients showed improved ORR [206,207].

#### 3.3.2. Talazoparib 

Talazoparib has the highest PARP-DNA trapping efficiency among the PARPis [208]. A phase II trial testing the effects of talazoparib on gBRCAm early-stage BC showed decreased tumor size in all patients included [209]. Other phase I and II trials with gBRCAm BC patients receiving talazoparib confirmed the efficiency of this PARPi [210,211]. The EMBRACA study, an open-label phase III trial, compared talazoparib monotherapy to chemotherapy in gBRCAm, HER2-negative BC patients [212]. PFS and ORR were improved with talazoparib compared to chemotherapy alone.

#### 3.3.3. Veliparib 

Veliparib has been mostly evaluated in combination with chemotherapy. For example, the phase II multicenter I-SPY2 trial tested the combination of veliparib and neoadjuvant chemotherapy in unselected TNBC patients [213]. The predicted complete response rate (pCR) was 51% with veliparib and chemotherapy vs. 26% in the control arm (chemotherapy alone). The phase II BROCADE study evaluated the combination of veliparib with carboplatin and paclitaxel in gBRCAm BC patients [214]. The ORR was improved with the combination of veliparib and chemotherapy compared to chemotherapy alone. Lastly, the phase III BRIGHTNESS study evaluated the addition of veliparib to carboplatin in the standard neoadjuvant chemotherapy setting [211]. The addition of veliparib showed no further benefit to chemotherapy.

#### 3.3.4. Rucaparib 

Rucaparib is the second PARPi that has been FDA approved for gBRCAm BC patients [215]. Intravenous rucaparib was tested in a phase II trial of gBRCAm BC patients [216]. Stable disease, meaning no tumor development, was reported in 44% of patients. Rucaparib was also tested in combination with chemotherapy in unselected TNBC patients [217]. This phase I study showed that rucaparib could be safely used in combination with chemotherapy. The phase II, a randomized BRE09-146 trial, evaluated rucaparib in combination with cisplatin vs. cisplatin alone in gBRCAm patients with residual disease following neoadjuvant therapy [218]. DFS was similar in the two arms, as low-dose rucaparib did not affect cisplatin toxicity. However, the rucaparib dose may not have been sufficient to inhibit PARP activity.

Tumor cells can become resistant to PARPi by different mechanisms [219]. 

First, secondary intragenic mutations that restore BRCA proteins functions can lead to PARPi resistance [220]. These genetic events can lead to the expression of nearly full-length proteins or full-length wild-type proteins with complete restored functions [221]. This has been reported mostly in ovarian cancer patients, and it has also been demonstrated in BC cell line models [222]. Tumor cells with missense mutations conserving the N-terminal and C-terminal domains of BRCA proteins also lead to poor PARPi response [223]. Another mechanism of action leading to PARPi resistance is decreased expression of PARP enzymes. Indeed, tumor cells with low PARP1 expression acquire resistance to veliparib [224]. 

In addition, tumor cells can find alternative mechanisms to protect the replication fork. It has been shown that PARPi-resistant cells can reduce the recruitment of the MRE11 (meiotic recombination 11) nuclease to the damage site, leading to the protection of the fork by blocking its access [225]. Another study has shown that *BRCA2*-mutated tumors acquired PARPi resistance by reducing the recruitment of the MUS81 (methyl methanesulfonate ultraviolet sensitive gene clone 81) nuclease to protect the replication fork [226]. 

Chemotherapy has been the pioneer treatment strategy for TNBC for decades. The development of PARPis has been a major improvement in the treatment of TNBC and, more specifically, gBRCAm TNBC, as they have shown more benefits over chemotherapy [227]. However, TNBC is a heterogenous BC subtype, and PARPis cannot treat all TNBCs as it is administered only for gBRCAm TNBC [228]. Therefore, it is necessary to develop specific targeted therapies to treat each TNBC subtype.

## 4. New Strategies and Challenges for Breast Cancer Treatment

### 4.1. Emerging Therapies for HR-Positive Breast Cancer

As mentioned in Section 3.1, the major mechanisms of action of current endocrine therapy resistance occur via (1) the mTOR/PI3K/Akt signaling pathway and (2) the actors of the cell cycle progression CDK4/6. Therefore, emerging therapies for HR+ BC mainly target these pathways to bypass estrogen-independent cell survival [229]. The most recent completed clinical trials on emerging therapies for HR+ BC are presented in Table 1.

#### 4.1.1. mTOR/PI3K/AKT Pathway Inhibitors 

The mTOR/PI3K/Akt pathway inhibitors can be divided into different categories according to the target in the pathway. Specific inhibitors have been developed to target all or specific isoforms of PI3K, mTORC1 and Akt [251]. 

##### Pan-Pi3K Inhibitors 

Pan-PI3K inhibitors target all PI3K isoforms resulting in significant off-target effects. The main pan-PI3K inhibitors are buparlisib and pictilisib [252]. Multiple clinical trials have tested the effects of pan-PI3K inhibitors in luminal BC. 

The phase III randomized double-blinded BELLE-2 trial compared buparlisib combined with fulvestrant, to fulvestrant monotherapy in luminal A advanced or metastatic BC patients [230]. The results of this trial showed a modest improvement in PFS when buparlisib was added to fulvestrant. Another phase III clinical trial (BELLE-3) studied the effects of buparlisib plus fulvestrant in luminal A advanced or metastatic BC patients with no benefits from endocrine therapy [231]. Though PFS was significantly improved with buparlisib, there were severe adverse effects such as hyperglycemia, dyspnea, or pleural effusion. Lastly, the phase II/III BELLE-4 clinical trial evaluated buparlisib plus paclitaxel in HER2-negative locally advanced or metastatic BC patients [232]. The addition of buparlisib to paclitaxel did not improve PFS in these patients. Thus, further studies on buparlisib in HR+ BC were not conducted. The phase II randomized, double-blinded FERGI clinical trial analyzed the effects of pictilisib plus fulvestrant in luminal A BC patients resistant to AI [233]. The addition of pictilisib to fulvestrant did not improve PFS. Moreover, severe adverse effects occurred when the dose of pictilisib was increased. These results were confirmed for pictilisib plus paclitaxel, as the phase II PEGGY study showed no benefit from pictilisib in PI3K-mutated HER2-negative BC patients [234].

Hence, pan-PI3K inhibitors are not optimal to treat HR+ BC due to their toxicity and lack of efficacy.

##### Isoform-Specific PI3K Inhibitors 

To sort out issues related to off-target effects and toxicities with pan-PI3K inhibitors, isoform-specific PI3K inhibitors have been developed. These isoform-specific PI3K inhibitors can specifically target the PI3K p110α, p110β, p110δ, and p110γ isoforms [252]. Multiple clinical trials have tested the effects of isoform-specific PI3K inhibitors. 

PI3K p110α is the most commonly mutated isoform in BC [253]. Alpelisib is the first FDA-approved PI3K p110α isoform inhibitor. A phase Ib clinical trial tested the effects of alpelisib and letrozole in patients with ER+ metastatic BC refractory to endocrine therapy [235]. The clinical benefit of the alpselisib and letrozole combination was higher for patients with PI3K-mutated BC, but clinical activity was still observed in patients with non-mutated tumors. The phase III randomized SOLAR-1 clinical trial compared the effects of alpelisib plus fulvestrant to fulvestrant alone in luminal A advanced BC patients who received no benefits from prior endocrine therapy [236]. The addition of alpelisib improved PFS for patients with PI3K-mutated BC. 

Taselisib targets the PI3K p110α, p110γ and p110δ isoforms [254]. Taselisib was tested in the SANDPIPER study, a phase III randomized clinical trial, in combination with fulvestrant in patients with ER+ metastatic BC resistant to AIs [238]. Although the addition of taselisib slightly improved PFS, further clinical trials with taselisib were interrupted since high rates of severe adverse events were detected. 

##### mTORC1 Inhibitors 

mTORC1 inhibitors, such as everolimus, block the mTORC1 dependent phosphorylation of s6k1 [255]. The BOLERO-2 phase III randomized clinical trial investigated the effects of exemestane with or without everolimus in AI-resistant ER+ metastatic BC patients [240]. The combination of everolimus and exemestane improved PFS. The TAMRAD phase II randomized open-label study compared the effects of tamoxifen with or without everolimus in AI-resistant luminal A BC patients [241]. This study showed an improvement in overall survival (OS) when everolimus was given in combination with tamoxifen. The findings of these two clinical trials led to FDA approval of everolimus. More recently, the PrE0102 phase II randomized clinical trial showed that the addition of everolimus to fulvestrant improved PFS of patients with AI-resistant ER+ BC compared to fulvestrant alone [242].

##### Akt Inhibitors 

Akt inhibitors target all Akt isoforms as Akt 1, 2, and 3 isoforms share very similar structures [256]. Capivasertib is the principal Akt inhibitor under investigation in different clinical trials. The FAKTION phase II multi-centered randomized clinical trial compared the effects of capivasertib plus fulvestrant to fulvestrant plus placebo in AI-resistant luminal A advanced BC patients [243]. PFS was significantly improved with the combination of capivasertib and fulvestrant in comparison with the placebo arm. 

The AKT1^E17K^ activating mutation is the most common in Akt and occurs in approximately 7% of ER+ metastatic BC. This mutation in the Akt lipid-binding pocket leads to constitutive Akt activation by modifying its localization to the membrane [257]. A phase I study analyzed the effects of capivasertib alone or in combination with fulvestrant in a cohort of patients with AKT1^E17K^ mutation ER+ metastatic BC [244]. Capivasertib, in combination with fulvestrant, demonstrated clinically meaningful activity and better tolerability compared to capivasertib alone. 

#### 4.1.2. CDK4/6 Inhibitors 

There are currently three CDK4/6 inhibitors approved to treat HR+/HER2- metastatic BC: palbociclib, ribociclib, and abemaciclib. They can be administered as first-line treatment combined with AIs or as second-line treatment combined with fulvestrant [258].

##### First-Line Treatment

Palbociclib, a highly selective CDK4/6 inhibitor, is the first FDA-approved CDK4/6 inhibitor as first-line treatment combined with AIs for metastatic or advanced HR+ BC patients [259]. 

PALOMA-1 is an open-label, randomized phase II study that evaluated the effects of palbociclib in combination with letrozole vs. letrozole alone as first-line treatment for HR+ advanced BC patients [126]. The addition of palbociclib to letrozole significantly improved PFS in HR+ BC patients. A phase III study was performed (PALOMA-2) to confirm these findings and expand the efficacy and safety of palbociclib, [245]. This double-blinded clinical trial tested the combination of palbociclib and letrozole in postmenopausal BC patients without prior systemic therapy for advanced BC. The addition of palbociclib to letrozole significantly improved PFS and ORR. 

Ribociclib is the second FDA-approved CDK4/6 inhibitor for first-line treatment in postmenopausal advanced BC patients in combination with AIs [260]. The phase III MONALEESA-2 clinical trial results showed improved PFS and ORR with the combination of ribociclib and letrozole in HR+ metastatic BC patients. The clinical benefits and manageable tolerability observed with ribociclib and letrozole are maintained with longer follow-up compared to letrozole alone [247]. 

Abemaciclib has been tested in the phase III randomized double-blinded MONARCH-3 study [250]. HR+ advanced BC patients with no prior systemic therapy received abemaciclib plus anastrozole or letrozole or AIs plus placebo in the control arm. PFS and ORR were significantly improved with the combination of abemaciclib and AIs. 

##### Second-Line Treatment 

As second-line treatment, palbociclib can be given in combination with fulvestrant in advanced or metastatic BC patients with disease progression after endocrine therapy [261]. This was confirmed in the phase III multi-centered randomized double-blinded PALOMA-3 trial [246]. BC patients who received palbociclib plus fulvestrant had significantly longer PFS compared to fulvestrant plus placebo. 

The phase III MONALEESA-3 study tested the effects of ribociclib plus fulvestrant in patients with HR+ advanced BC who received prior endocrine therapy in the advanced setting [248]. The PFS and ORR were significantly improved when ribociclib was added to fulvestrant. Thus, ribociclib plus fulvestrant can be considered as second-line treatment for these BC patients. 

Abemaciclib has been recently approved in combination with fulvestrant for HR+ advanced or metastatic BC patients with disease progression after endocrine therapy. This was based on the results of the phase III, double-blinded MONARCH 2 study [249]. The combination of abemaciclib and fulvestrant demonstrated a significant improvement of PFS and ORR compared to fulvestrant plus placebo in HR+ metastatic BC patients who experienced relapse or progression after prior endocrine therapy. 

mTOR/PI3K/Akt inhibitors and CDK4/6 inhibitors show great promise for advanced HR+ BC resistant to endocrine therapy. To leverage the potential of these two types of therapies, some preclinical studies have evaluated a triple therapy combination including PI3K inhibitors, CDK4/6 inhibitors, and endocrine therapy (see the summarized table at the end of the manuscript) [262].

### 4.2. New Strategic Therapies for HER2-Positive Breast Cancer 

As mentioned in Section 3.2, HER2+ BC is currently treated with specific HER2 targeting antibodies or tyrosine kinase inhibitors (TKIs), and more recently, with TDM-1, an antibody-drug conjugate. These treatments have greatly improved HER2+ BC survival. However, 25% of HER2+ BC patients will still develop resistance to anti-HER2 treatment. Hence, new therapeutic strategies are emerging, such as new antibodies targeting HER2, new TKIs, vaccines, and PI3K/mTOR and CDK4/6 inhibitors [263]. The most recent completed clinical trials on new strategies for HER2+ BC treatment are gathered in Table 2.

#### 4.2.1. New Antibodies 

Novel types of antibodies have been developed to target HER2+ BC more efficiently. They can be divided into three categories: antibody-drug conjugates (ADC), modified antibodies, and bispecific antibodies.

##### Antibody-Drug Conjugates (ADC)

ADCs are the combination of a specific monoclonal antibody and a cytotoxic drug that is released in the antigen-expressing cells [280]. The most common ADC is TDM-1, and the promising results with TDM-1 have led to the development of new ADCs. 

Trastuzumab-deruxtecan (DS-8201a) is a HER2-targeting antibody (trastuzumab) linked to a DNA topoisomerase I inhibitor (deruxtecan) [281]. A phase I study demonstrated that DS-8201a had antitumor activity even with HER2 low-expressing tumors [282]. These results led to phase II and phase III clinical trials. The DESTINY-Breast01 clinical trial is an open-labeled, single-group, multicentered phase II study [264] was evaluated in HER2+ metastatic BC patients who received prior TDM-1 treatment. DS-8201a showed durable antitumor activity for these patients. Two phase III clinical trials are currently evaluating DS-8201a. DESTINY-Breast02 (ClinicalTrials.gov identifier: NCT03523585) is comparing DS-8201a to standard treatment (lapatinib or trastuzumab) in HER2+ metastatic BC patients previously treated with TDM-1. The DESTINY-Breast03 (ClinicalTrials.gov identifier: NCT03529110) trial is evaluating the effects of DS-8201a vs. TDM-1 in HER2+ metastatic BC patients with prior trastuzumab and taxane treatment. 

Trastuzumab-duocarmycin (SYD985) is a HER-2 targeting antibody (trastuzumab) conjugate with a cleavable linker-duocarmycin payload that causes irreversible alkylation of the DNA in tumor cells leading to cell death [283]. A dose-escalation phase I study evaluated the effects of SYD85 in BC patients with variable HER2 status and refractory to standard cancer treatment [284]. Trastuzumab-duocarmycin showed clinical activity in heavily pretreated HER2+ metastatic BC patients, including TDM-1 resistant and HER2-low BC patients. After these promising results, a phase I expansion cohort study was performed on the same type of patients (heavily pretreated HER2+ or HER2-low BC patients) [265]. This study confirmed previous results on the efficacy of STD985. A phase III clinical trial (TULIP-ClinicalTrials.gov identifier: NCT03262935) is ongoing to compare SYD985 to the treatment chosen by the physician in HER2+ metastatic BC patients. Other ADCs are under clinical trials to test their safety and activity for HER2+ advanced BC patients. RC48 is an anti-HER2 antibody conjugated with monomethyl auristatin E that demonstrated promising efficacy and a manageable safety profile in an open-labeled, multicentered phase II study (ClinicalTrials.gov identifier: NCT02881138) [248]. PF06804103 conjugates an anti-HER2 monoclonal antibody and the cytotoxic agent, Aur0101. In a phase I study (ClinicalTrials.gov identifier: NCT03284723), PF06804103 showed manageable toxicity and promising antitumor activity [249]. XMT1522 showed encouraging results in a dose-escalation phase I study (ClinicalTrials.gov identifier: NCT02952729) [250]. MEDI4276, which targets two different HER2 epitopes and is linked to a microtubule inhibitor, showed promising clinical activity in a phase I study (ClinicalTrials.gov identifier: NCT02576548) [254] (see the summarized table at the end of the manuscript).

##### Chimeric Antibody 

Margetuxumab (MGAH22) is a human/mouse chimeric IgG1 targeting HER2 monoclonal antibody. It is based on trastuzumab as it binds to the same epitope (subdomain IV or HER2 extracellular domain) but with an enhanced Fcγ domain. The substitution of five amino acids into the IgG1 Fc domain increases CD16A affinity, a receptor found on macrophages and natural-killer cells, and decreases CD32B affinity, leading to increased antibody-dependent cell-mediated cytotoxicity (ADCC) [285]. A phase I study evaluated margetuximab toxicity and tumor activity on HER2+ BC patients for whom no standard treatment was available [266]. This study showed promising single-agent activity of margetuximab as well as good tolerability. The phase III randomized open-labeled SOPHIA clinical trial (ClinicalTrials.gov Identifier: NCT02492711) compared margetuximab plus chemotherapy vs. trastuzumab plus chemotherapy in pretreated HER2+ advanced BC patients [286]. The combination of margetuximab and chemotherapy significantly improved the PFS of patients compared to trastuzumab plus chemotherapy. This study is still under investigation to collect data on OS (see the summarized table at the end of the manuscript).

##### Bispecific Antibodies 

Bispecific antibodies (BsAbs) can target two different epitopes in the same or different receptors by combining the functionality of two monoclonal antibodies [287]. MCLA-128 targets both HER2 and HER3 and have an enhanced ADCC activity [288]. A phase I/II study evaluated the safety, tolerability, and antitumor activity of MCLA-128 in patients with pretreated HER2+ metastatic BC.

Preliminary results showed encouraging clinical benefits of MCLA-128. An open-labeled, multicentered phase II study (ClinicalTrials.gov identifier: NCT03321981) is ongoing to evaluate the effects of MCLA-128 in combination with trastuzumab and chemotherapy in HER2+ metastatic BC patients. 

ZW25 is a BsAb biparatopic that binds the IV and II subdomains of the HER2 extracellular domain, the binding epitopes of trastuzumab and pertuzumab, respectively [289]. The efficacy of ZW25 was evaluated in a phase I study given alone or in combination with chemotherapy in patients with advanced or metastatic HER2+ BC. The results of this study showed promising antitumor activity, and no-dose limiting was observed. 

T-cell bispecific antibodies (TCBs) are another type of BsAbs recently developed. TCBs target the CD3-chain of the T-cell receptor and tumor-specific antigens, resulting in lymphocyte activation and tumor cell death [290]. 

GBR1302 targets both HER2 and CD3 receptors and directs T-cells to HER2+ tumor cells. A phase II study (ClinicalTrials.gov identifier: NCT03983395) is ongoing to determine the safety profile of the GBR1302 single agent in previously treated HER2+ metastatic BC. PRS-343 targets HER2 and the immune receptor CD137, a member of the tumor necrosis factor receptor family. Two clinical trials are investigating the effects of PRS-343 monotherapy (ClinicalTrials.gov identifier: NCT03330561) or in combination with other treatments (ClinicalTrials.gov identifier: NCT03650348) (see the summarized table at the end of the manuscript).

#### 4.2.2. HER2-Derived Peptide Vaccines 

One of the strategies of immunotherapy is activating the patient’s immune system to kill cancer cells. Vaccination is an emerging approach to induce a tumor-specific immune response by targeting tumor-associated antigens, such as HER2 [291]. HER2-derived peptide vaccines comprise different parts of the HER2 molecule, such as E75 (extracellular domain), GP2 (transmembrane domain), and AE37 (intracellular domain) [292].

E75 (HER2/neu 369–377: KIFGSLAFL) has high affinity for HLA2 and HLA3 (human leucocyte antigen) that can stimulate T-cells against HER2 overexpressing tumor cells [293]. The efficacy of the E75 vaccine to prevent BC recurrence has been evaluated in a phase I/II study, in which high-risk HER2+ HLA2/3+ BC patients received the E75 vaccine [269]. The results demonstrated the safety and clinical efficacy of the vaccine as PFS was improved in the E75-vaccinated group compared to the unvaccinated group. Other clinical trials are currently investigating the efficacy of the E75 vaccine on HER2+ BC (see he summarized table at the end of the manuscript). 

GP2 (654-662: IISAVVGIL) is a subdominant epitope with poor affinity for HLA2 [294]. A phase I trial evaluating the effects of a GP2 vaccine in disease-free BC patients showed that it was safe and tolerable with HER2-specific immune response [295]. The GP2 vaccine has been tested in a randomized, open-labeled phase II study to prevent BC recurrence. The patients that received the GP2 vaccine had HER2+ and HLA2+ BC and were disease-free with a high risk of recurrence at the time of the study [270]. The results demonstrated that the GP2 vaccine was safe and clinically beneficial for patients with HER2+ BC who received the full vaccine series. 

AE37 (Ii-key hybrid of MHC II peptide AE36 (HER2/neu 776–790)) can stimulate CD8+ and CD4+ cells. A randomized, single-blinded phase II study evaluated the effects of an AE37 vaccine to prevent BC recurrence. Patients with a high risk of recurrence and HER2+ BC received the AE37 vaccine [271]. The vaccination demonstrated no benefit in the overall intention-to-treat analysis, a method that considers the randomized treatment to avoid bias happening after the randomization [296]. However, the study showed that the AE37 vaccine was safe, and results suggested that it could be effective for HER2-low BC, such as TNBC.

#### 4.2.3. New Tyrosine Kinase Inhibitors (TKIs)

As previously described in this review (see Section 3.2.2 Tyrosine kinase inhibitors (TKIs)), TKIs are small molecules targeting the HER2 intracellular catalytic domain [159]. New TKIs have been developed with better efficacy and less toxicity in the treatment of HER2+ metastatic BC, such as tucatinib and poziotinib. 

Tucatinib is a TKI with high selectivity for HER2, leading to less EGFR-related toxicities, common with other HER TKIs [297]. A phase I dose-escalation trial evaluated the combination of tucatinib and trastuzumab in BC patients with progressive HER2+ brain metastases [298]. This study showed preliminary evidence of tucatinib efficacy and tolerability in these patients. Tucatinib was also tested in combination with TDM-1 in a phase Ib trial in HER2+ metastatic BC patients with heavy pre-treatment [299]. The combination of tucatinib and TDM-1 showed acceptable toxicity and antitumor activity in these patients. Tucatinib was FDA approved in combination with trastuzumab and capecitabine for patients with advanced or metastatic HER2+ BC who received prior anti-HER2 in the metastatic setting [300]. This was based on the results of the phase II HER2CLIMB clinical trial, where HER2+ metastatic BC patients received tucatinib or placebo in combination with trastuzumab and capecitabine [267]. The addition of tucatinib to trastuzumab and capecitabine improved PFS and OS of heavily pretreated HER2+ metastatic BC patients. 

Poziotinib is a pan-HER kinase inhibitor that irreversibly inhibits the HER family members’ kinase activity [301]. A phase I study evaluated the efficacy and tolerability of poziotinib in advanced solid tumors. The results showed encouraging antitumor activity against different types of HER2+ cancers as poziotinib was safe and well-tolerated by the patients [302]. The phase II NOV120101-203 study evaluated the safety and efficacy of poziotinib monotherapy in heavily pretreated HER2+ metastatic BC patients [268]. Poziotinib showed meaningful activity in these patients with no severe toxicities. 

#### 4.2.4. mTOR/PI3K Inhibitors and CDK4/6 Inhibitors 

As mentioned in the previous Section 4.1, mTOR/PI3K inhibitors and CDK4/6 inhibitors have been evaluated as potential new strategic therapies for HR+ BC resistant to endocrine therapy. The mTOR/PI3K signaling pathway and CDK4/6 also play a role in the mechanisms leading to treatment resistance in HER2+ BC [303]. Thus, targeting them with mTOR/PI3K and CDK4/6 inhibitors is also being investigated in HER2+ BC subtype.

##### mTOR/PI3K Inhibitors 

Alpelisib and taselisib are PI3K isoform-specific inhibitors that were also evaluated in HR+ BC [235,236,238,253,254]. A phase I study evaluated alpelisib in combination with trastuzumab and LJM716 (a HER3-targeted antibody) in patients with PI3KCA mutant HER2+ metastatic BC [272]. Unfortunately, the results of this study were limited by high gastrointestinal toxicity. Another phase I study tested alpelisib in combination with TDM-1 in HER2+ metastatic BC patients pretreated with trastuzumab [273]. The combination of alpelisib and TDM-1 demonstrated tolerability and antitumor activity in trastuzumab-resistant HER2+ metastatic BC patients. Taselisib is being tested in an ongoing phase Ib dose-escalation trial in combination with anti-HER2 therapies (trastuzumab, pertuzumab and TDM-1) in HER2+ advanced BC patients (ClinicalTrials.gov identifier: NCT02390427). 

Copanlisib is a highly selective and potent pan-class I PI3K inhibitor [304]. A phase Ib (PantHER) study evaluated the tolerability and activity of copanlisib in combination with trastuzumab in heavily pretreated HER2+ metastatic BC patients [274]. The combination of copanlisib and trastuzumab was safe and tolerable. Preliminary evidence of tumor stability was observed in these patients. 

Everolimus is a mTORC1 inhibitor also tested in HR+ BC [240,241,242]. Everolimus was tested in phase III clinical trials, in combination with trastuzumab and docetaxel (BOLERO-1), or in combination with trastuzumab and vinorelbine (BOLERO-3) in trastuzumab-resistant advanced HER2+ BC [275,276]. Unfortunately, results showed an increase of adverse effects with everolimus. Moreover, the BOLERO-1 clinical trial showed no improvement in PFS with the combination of trastuzumab and everolimus. By contrast, PFS was significantly longer when everolimus was added to vinorelbine in BOLERO-3. A study analyzing the molecular alterations found in patients in the BOLERO-1 and BOLERO-3 clinical trials demonstrated that HER2+ BC patients could derive more benefit from everolimus if the tumors had PI3KCA mutations, PTEN loss or a hyperactive PI3K pathway [305]. 

##### CDK4/6 Inhibitors 

Palbociclib, ribociclib and abemaciclib are CDK4/6 inhibitors that have been FDA approved to treat HR+ BC as first-line treatments [247,250,259]. They have also been evaluated in multiple clinical trials for advanced HER2+ BC. Palbociclib has been tested in combination with trastuzumab in the phase II SOLTI-1303 PATRICIA clinical trial in heavily pretreated advanced HER2+ BC patients [277]. Palbociclib combined with trastuzumab demonstrated safety and encouraging survival outcomes in these patients. Palbociclib has also been evaluated in combination with TDM-1 in HER2+ advanced BC patients pretreated with trastuzumab and taxane therapy [306]. The results of this phase I/Ib study showed safety, tolerability, and antitumor activity in these patients. 

Ribociclib was evaluated in a phase Ib/II trial in combination with trastuzumab to treat advanced HER2+ BC patients previously treated with multiple anti-HER2 therapies [278]. The combination of ribociclib and trastuzumab was safe, but there was limited activity in heavily pretreated patients. The conclusions of this study suggest that CDK4/6 inhibitor/anti-HER2 combination should be administered in patients with few previous therapies. 

Abemaciclib has been tested in the phase II randomized open-labeled MonarcHER trial in combination with trastuzumab with or without fulvestrant vs. trastuzumab with standard chemotherapy in HR+/HER2+ BC patients [279]. The combination of abemaciclib, trastuzumab, and fulvestrant significantly improved PFS in these patients, with a tolerable safety profile. 

There are multiple ongoing clinical trials for advanced HER2+ BC testing the combination of palbociclib, trastuzumab, pertuzumab, and anastrozole (ClinicalTrials.gov identifier: NCT03304080); or palbociclib and trastuzumab plus letrozole (ClinicalTrials.gov identifier: NCT03054363). Preliminary results are expected around July 2021 and March 2022, respectively (see he summarized table at the end of the manuscript). 

A great proportion of HER2+ BC patients develop resistance to traditional anti-HER2 therapies, and 40–50% of patients with advanced HER2+ BC develop brain metastases [307]. Thus, developing new therapies to overcome resistance is essential. The therapeutic strategies that have been described in this section provide new hope for HER2+ BC patients, especially for advanced or metastatic HER2+ BC patients. 

### 4.3. Emerging Therapies for Triple Negative Breast Cancer (TNBC)

TNBC is the most aggressive BC subtype. The fact that TNBC lacks ER and PR expression and does not overexpress HER2, combined with its high heterogeneity, has contributed to the difficulties in developing efficient therapies [308]. Thus, multiple strategic therapies have been developed to treat all TNBC subtypes. These include conjugated antibodies, targeted therapy, and immunotherapy. An overview of the most recent and completed clinical trials on emerging therapies for TNBC is presented in Table 3.

#### 4.3.1. Antibodies-Drug Conjugates (ADC)

Antibody drug conjugates (ADCs) deliver a cytotoxic drug into the tumor cell by the specific binding of an antibody to a surface molecule [280]. Multiple ADCs have been investigated in TNBC such as sacituzumab govitecan, ladiratuzumab vedotin, or trastuzumab deruxtecan. 

Sacituzumab govitecan combines an antibody targeting trophoblast antigen 2 (Trop-2) and a topoisomerase I inhibitor SN-38 [334]. Trop-2, a CA^2+^ signal transducer, is expressed in 90% of TNBCs and is associated with poor prognosis [335,336]. A single-arm, multicentered phase I/II study evaluated sacituzumab govitecan in heavily pretreated metastatic TNBC patients [336,337]. The efficacy and safety of scituzumab govitecan was shown in these patients, as it was associated with durable objective response. Based on these results, a randomized phase III trial (ASCENT) tested sacituzumab govitecan compared to single-agent chemotherapy chosen by the physician in patients with relapsed or refractory metastatic TNBC [309]. Sacituzumab govitecan significantly improved PFS and OS of metastatic TNBC patients compared to chemotherapy. 

Ladiratuzumab vedotin is composed of a monoclonal antibody targeting the zinc transporter LIV-1 and a potent microtubule disrupting agent, monoethyl auristatin E (MMAE) [338]. LIV-1 is a transmembrane protein with potent zinc transporter and metalloproteinase activity, expressed in more than 70% of metastatic TNBC tumors [339]. All clinical trials investigating ladiratuzumab vedotin are still ongoing. A dose-escalation phase I study is evaluating the safety and efficacy of ladiratuzumab vedotin in heavily pretreated metastatic TNBC patients (ClinicalTrials.gov identifier: NCT01969643). Preliminary results showed encouraging antitumor activity and tolerability of ladiratuzumab vedotin with an objective response rate of 32% [340]. The estimated study completion date is June 2023. Two phase Ib/II trials are testing ladiratuzumab vedotin in combination with immunotherapy agents in metastatic TNBC patients, such as pembrolizumab (ClinicalTrials.gov Identifier: NCT03310957) with expected preliminary results in February 2022, or in combination with multiple immunotherapy-based treatments (ClinicalTrials.gov Identifier: NCT03424005) with expected preliminary results in January 2023. 

Trastuzumab deruxtecan is an ADC developed as a treatment for metastatic HER2+ BC patients. Its mechanism of action is described in Section 3.2. Even though trastuzumab deruxtecan was developed to treat HER2+ BC, it showed antitumor activity in HER2-low tumors in a phase I study [282]. Based on these results, an ongoing open-labeled, multicentered phase III study (ClinicalTrials.gov Identifier: NCT03734029) is recruiting patients with HER2-low metastatic BC to test trastuzumab deruxtecan vs. standard treatment chosen by the physician. Preliminary results are expected in January 2023 (see Table 4). 

#### 4.3.2. Targeted Therapies 

Targeted therapy is the current standard of care to treat HR+ and HER2+ BC, but it cannot be administered to patients with TNBC as these tumors lack the expression of these biomarkers. Hence, the next logical step is to identify biomarkers associated with TNBC to develop specific targeted therapies. Several emerging targeted therapies are being clinically trialed with limited or mixed results.

##### VEGF and EGFR Inhibitors 

Vascular endothelial growth factor (VEGF) and epidermal growth factor receptor (EGFR) are overexpressed in most TNBC patients [341,342]. Bevacizumab and cetuximab are antibodies developed to specifically target VEGF and EGFR, respectively. Unfortunately, clinical trials studying the effects of these antibodies in TNBC patients demonstrated limited results. The phase III, randomized BEATRICE study evaluating adjuvant bevacizumab-continuing therapy in TNBC demonstrated no significant benefit in OS [310]. A phase II trial evaluating the impact of adding bevacizumab or cisplatin to neoadjuvant chemotherapy to stage II to III TNBC concluded that further investigation of bevacizumab in this setting was unlikely [311]. 

The phase II randomized TBCRC 001 trial testing the combination of cetuximab and carboplatin in stage IV TNBC showed a response in fewer than 20% of patients [312]. Another randomized phase II study compared the effects of cetuximab plus cisplatin to cisplatin alone in metastatic TNBC patients. Adding cetuximab to cisplatin prolonged PFS and OS, warranting further investigation of cetuximab in TNBC [313]. Based on these results, bevacizumab is not recommended for the treatment of TNBC. 

##### mTOR/PI3K/AKT Inhibitors 

mTOR/PI3K/Akt signaling pathway is an important target involving all BC subtypes. Inhibitors of mTOR, PI3K, and Akt have been tested in HR+ and HER2+ BC patients and have also been tested in TNBC patients. The mTOR inhibitor everolimus has been tested in a randomized phase II trial in combination with chemotherapy vs. chemotherapy alone in stage II/III TNBC patients [314]. Unfortunately, the addition of everolimus was associated with more adverse effects, without improving pCR or clinical response. A phase I study testing the combination of everolimus and eribulin in metastatic TNBC patients showed that this combination was safe, but the efficacy was modest [343]. 

The Akt inhibitor ipatasertib has been tested in combination with paclitaxel (vs. placebo) for metastatic TNBC patients in the phase II multicentered double-blinded randomized LOTUS trial [315]. The results showed improved PFS when patients received ipatasertib. Another phase II double-blinded randomized trial, FAIRLANE, testing neoadjuvant ipatasertib plus paclitaxel for early TNBC, showed no clinically or statistically significant improvement in the pCR rate, but ipatasertib’s antitumor effect was more pronounced in patients with PI3K/AKT1/PTEN-altered tumors [316]. Capivasertib, another Akt inhibitor, has been tested in combination with paclitaxel (vs. placebo), first-line therapy for metastatic TNBC patients in the phase II double-blinded randomized PAKT trial [317]. The addition of capivasertib to paclitaxel significantly improved PFS and OS, with better benefits for patients with PI3K/AKT1/PTEN-altered tumors. 

##### Androgen Receptor Inhibitors 

The androgen receptor (AR) is a steroidal hormonal receptor that belongs to the nuclear receptor family and is expressed in 10% to 50% of TNBC tumors [344]. Tumors expressing AR have better prognosis but are less responsive to chemotherapy [345]. Multiple clinical trials have tested AR inhibitors in TNBC [318,319,320]. 

Bicalutamide, an AR agonist, was tested in a phase II study in patients with AR+, HR- metastatic BC [318]. The results showed promising efficacy and safety for these patients. 

Enzalutamide, a nonsteroidal antiandrogen, has been tested in a phase II study in patients with locally advanced or metastatic AR+ TNBC [319]. Enzalutamide demonstrated significant clinical activity and tolerability, warranting further investigation. 

Abiraterone, a selective inhibitor of CYP17, has been evaluated in combination with prednisone in AR+ locally advanced or metastatic TNBC patients [320]. This combination was beneficial for 20% of the patients. 

Several clinical trials are currently testing AR inhibitors alone or combined with other treatments for TNBC patients; expecting results between 2022 and 2027 (see Table 4). 

#### 4.3.3. Immunotherapy 

##### Targeted Antibodies 

The immune system plays a crucial role in BC development and progression. Tumor cells can escape the immune system by regulating T-cell activity leading to the inhibition of immune response [346,347]. Two principal biomarkers found in TNBC are associated with this bypass: the programmed cell death protein receptor (PD-1) and its ligand PDL-1, and the cytotoxic T lymphocyte-associated protein 4 (CTLA-4) [348]. 

PD-1 is an immune checkpoint receptor expressed on the surface of activated T-cells. PDL-1, its ligand, is expressed on the surface of dendritic cells or macrophages. The interaction of PD-1 and PDL-1 inhibits T-cell response [349]. CTLA-4 is expressed on T-cells and inhibits T-cell activation by binding to CD80/CD86, leading to decreased immune response [350]. 

Atezolizumab, an anti-PDL-1 antibody, has demonstrated safety and efficacy in a phase I study for metastatic TNBC patients [351]. Based on these results, atezolizumab was tested in combination with nab-paclitaxel for unresectable locally advanced or metastatic TNBC in the phase III double-blinded placebo-controlled randomized Impassion130 study [321]. Atezolizumab plus nab-paclitaxel prolonged PFS and OS in both the intention-to-treat population and PDL1+ subgroup. Another double-blinded, randomized phase III study (Impassion031) compared atezolizumab in combination with nab-paclitaxel and anthracycline-based chemotherapy vs. placebo for early-stage TNBC [322]. This combination significantly improved pCR with an acceptable safety profile. 

Durvalumab, another anti-PDL-1 antibody, has been tested in combination with an anthracycline taxane-based neoadjuvant therapy for early TNBC in the randomized phase II GeparNuevo study [323]. This combination increased pCR rate, particularly in patients pretreated with durvalumab monotherapy before chemotherapy. Another randomized phase II study, SAFIRO BREAST-IMMUNO, compared durvalumab to maintenance chemotherapy in a cohort including TNBC patients [324]. Results showed that durvalumab, as a single agent therapy, could improve outcomes in TNBC patients. A phase I study tested durvalumab in combination with multiple TNBC therapies: PARP inhibitor olaparib and VEGFR1-3 inhibitor cediranib for patients with recurrent cancers including TNBC [325]. This combination was well tolerated and showed preliminary antitumor activity in all of these patients. 

The safety and efficacy of avelumab, another anti-PDL-1 antibody, was evaluated in the phase Ib JAVELIN study in patients with locally advanced or metastatic BC, including TNBC [326]. Avelumab showed an acceptable safety profile and clinical activity, particularly in tumors expressing PDL-1. 

Pembrolizumab is an anti-PD-1 antibody that has been tested in multiple clinical trials. The phase Ib KEYNOTE-012 study demonstrated the safety and efficacy of pembrolizumab on advanced TNBC patients [352]. Based on these results, the phase II KEYNOTE-086 study evaluated pembrolizumab monotherapy for pretreated or non-pretreated metastatic TNBC patients [327,353]. Pembrolizumab monotherapy showed a manageable safety profile and durable antitumor activity for both pretreated and non-pretreated subgroups. The randomized open-labeled phase III KEYNOTE-119 trial compared pembrolizumab monotherapy to standard chemotherapy in metastatic TNBC [354]. Pembrolizumab monotherapy did not significantly improve OS compared to chemotherapy in these patients. These findings suggest that pembrolizumab should be investigated in a combinational approach rather than in monotherapy. Based on these results, pembrolizumab was tested in combination with chemotherapy (vs. placebo) for pretreated locally recurrent or metastatic TNBC patients in the phase III double-blinded randomized KEYNOTE-355 trial [328]. The combination of pembrolizumab plus chemotherapy significantly and clinically improved PFS compared to chemotherapy plus placebo. Pembrolizumab has also been evaluated for early TNBC as neoadjuvant therapy in combination with chemotherapy (vs. placebo) in the phase III KEYNOTE-522 trial [329]. The combination of pembrolizumab plus chemotherapy significantly improved pCR rate in these patients compared to placebo plus chemotherapy. 

Tremelimumab is an anti-CTLA-4 antibody. A dose-escalation phase I study evaluating the safety and efficacy of tremelimumab in patients with metastatic BC showed good tolerability [330]. 

##### Vaccines 

Vaccination is an emerging approach to prevent recurrence in high-risk BC patients. As mentioned earlier, TNBC is the most aggressive BC subtype with a higher risk of distant recurrence [331]. Thus, developing vaccines to prevent recurrence in TNBC patients is of great interest. 

Takahashi et al. have developed a novel regimen of personalized peptide vaccination (PPV) based on the patient’s immune system to select vaccine antigens from a pool of peptide candidates [332]. They performed a phase II study where metastatic recurrent BC patients with prior chemotherapy and/or hormonal therapies received a series of personalized vaccines. This vaccination demonstrated safety, possible clinical benefit, and immune response, especially for TNBC patients [332]. A multicentered, randomized, double-blinded phase III study analyzed the effects of sialyl-TN keyhole limpet hemocyanin (STn-KLH) on metastatic BC patients [333]. STn-KLH consists of a synthetic STn, an epitope expressed in BC and associated with aggressive and metastatic tumors, and a high molecular weight protein carrier KLH [355]. Stn-KLH demonstrated good tolerability, but no benefits in time to progression (TTP) or survival were found. Thus, this vaccination is not recommended for metastatic BC patients [333]. 

PVX-410 is a multiple peptide vaccine that activates T-cell to target tumor cells and was developed to treat myeloma. A phase Ib/II study demonstrated the safety and immunogenicity in myeloma patients [356]. Based on these results, a PVX-410 vaccine is currently being tested to treat TNBC in multiple clinical trials (see Table 4). 

Finding new treatments for TNBC is an ongoing challenge. The therapeutic strategies that have been described in this section offer great hope to treat TNBC patients. However, because TNBC is highly heterogeneous, it is difficult to find a single treatment efficient for all TNBC subtypes [228].

## 5. Conclusions 

This review clearly demonstrates that the treatment of BC is complex and is constantly evolving with a large number of ongoing clinical trials on emerging therapies. Indeed, the BC molecular subtype will determine the personalized therapeutic approach, such as targeted treatments like endocrine therapy for HR+ BC or anti-HER2 therapy for HER2+ BC. These therapies have demonstrated their safety and efficacy in treating BC over the years. However, it is essential to go beyond these conventional treatments as BC is a complex disease and not all patients can benefit from personalized treatment. One of the major challenges in BC treatment is finding effective therapies to treat TNBC patients since conventional targeted therapies cannot be administered for this specific BC subtype, which has the worst survival outcomes. 

Another important issue in BC treatment is the acquisition of treatment resistance. This is a common phenomenon for either endocrine therapy, anti-HER2 therapy, and chemotherapy.

Hence, understanding the mechanisms underlying drug resistance is a good strategy to develop novel treatments for BC. For example, the mTOR/PI3K/Akt pathway is involved in the mechanism of resistance in all BC molecular subtypes, and thus developing specific inhibitors targeting this pathway is a promising BC treatment approach. 

## Figures and Tables

**Table 1:** Most recent completed clinical trial on emerging therapies for HR-positive breast cancer.

Targeted Therapy	Drug Name	Trial Number	Patient Population	Trial Arms	Outcomes
Pan-PI3K inhibitors	Buparlisib	BELLE-2 Phase III NCT01610284 [230]	HR+/HER2- Postmenopausal Locally advanced or MBC Prior AI treatment	Buparlisib + fulvestrant vs. placebo + fulvestrant	PFS 6.9 months vs. 5.0 months (HR 0.78; *p* = 0.00021) PFS 6.8 months vs. 4.0 months in PI3K mutated (HR 0.76; *p* = 0.014)
		BELLE-3 Phase III NCT01633060 [231]	HR+/HER2- Postmenopausal Locally advanced or MBC Prior endocrine therapy or mTOR inhibitors	Buparlisib + fulvestrant vs. placebo + fulvestrant	PFS 3.9 months vs. 1.8 months (HR 0.67; *p* = 0.0003)
		BELLE-4 Phase II/III NCT01572727 [232]	HER2- Locally advanced or MBC No prior chemotherapy	Buparlisib + pacliatxel vs. placebo + paclitaxel	PFS 8.0 months vs. 9.2 months (HR 1.18, 95% CI 0.82–1.68) PFS 9.1 months vs. 9.2 months in PI3K mutated (HR 1.17, 95% 0.63–2.17)
	Pictilisib	FERGI Phase II NCT01437566 [233]	HR+/HER2- Postmenopausal Prior AI treatment	Pictilisib + fulvestrant vs. placebo + fulvestrant	PFS 6.6 months vs. 5.1 months (HR 0.74; *p* = 0.096) PFS 6.5 months vs. 5.1 months in PI3K mutated (HR 0.74; *p* = 0.268) PFS 5.8 months vs. 3.6 months in non-PI3K mutated (HR 0.72; *p* = 0.23)
		PEGGY Phase II NCT01740336 [234]	HR+/HER2- Locally recurrent or MBC	Pictilisib + paclitaxel vs. placebo + paclitaxel	PFS 8.2 months vs. 7.8 months (HR 0.95; *p* = 0.83) PFS 7.3 months vs. 5.8 months in PI3K mutated (HR 1.06; *p* = 0.88)
Isoform-specific inhibitors	Alpelisib	Phase Ib NCT01791478 [235]	HR+/HER2- Postmenopausal MBC Prior endocrine therapy	Alpelisib + letrozole	CBR 35% (44% in patients with *PIK3CA* mutated and 20% in *PIK3CA* wild-type tumors; 95% CI [17%; 56%])
		SOLAR-1 Phase III NCT02437318 [236]	HR+/HER2- Advanced BC Prior endocrine therapy	Alpelisib + fulvestrant vs. placebo + fulvestrant	PFS 7.4 months vs. 5.6 months in non-PI3K mutated (HR 0.85, 95% CI 0.58–1.25) PFS 11.0 months vs. 5.7 months in PI3K mutated (HR 0.65; *p* = 0.00065)
		NEO-ORB Phase II NCT01923168 [237]	HR+/HER2- Postmenopausal Early-stage BC Neoadjuvant setting	Alpelisib + letrozole vs. placebo + letrozole	ORR 43% vs. 45% (*PIK3CA* mutant), 63% vs. 61% (*PIK3CA* wildtype) pCR rates low in all groups
	Taselisib	SANDPIPER Phase III NCT02340221 [238]	HR+/HER2- Postmenopausal Locally advanced or MBC PIK3CA-mutant Prior AI treatment	Taselisib + fulvestrant vs. placebo + fulvestrant	PFS 7.4 months vs. 5.4 months (HR 0.70; *p* = 0.0037)
		LORELEI Phase II NCT02273973 [239]	HR+/HER2- Postmenopausal Early-stage BC Neoadjuvant setting	Taselisib + letrozole vs. placebo + letrozole	ORR 50% vs. 39.3% (OR 1.55; *p* = 0.049) ORR 56.2% vs. 38% in PI3K mutated (OR 2.03; *p* = 0.033) No significant difference in pCR
mTOR inhibitors	Everolimus	BOLERO-2 Phase III NCT00863655 [240]	HR+/HER2- Advanced BC Prior AI treatment	Everolimus + exemestane vs. placebo + exemestane	PFS 6.9 months vs. 2.8 months (HR 0.43; *p* < 0.001)
		TAMRAD Phase II NCT01298713 [241]	HR+/HER2- Postmenopausal MBC Prior AI treatment	Everolimus + tamoxifen vs. tamoxifen alone	CBR 61% vs. 42% TTP 8.6 months vs. 4.5 months (HR 0.54)
		PrE0102 Phase II NCT01797120 [242]	HR+/HER2- Postmenopausal MBC Prior AI treatment	Everolimus + fulvestrant vs. placebo + fulvestrant	PFS 10.3 months vs. 5.1 months (HR 0.61; *p* = 0.02) CBR 63.6% vs. 41.5% (*p* = 0.01)
Akt inhibitors	Capivasertib	FAKTION Phase II NCT01992952 [243]	HR+/HER2- Postmenopausal Locally advanced or MBC Prior AI treatment	Capivasertib + fulvestrant vs. placebo + fulvestrant	PFS 10.3 months vs. 4.8 months (HR 0.57; *p* = 0.0035)
		Phase I NCT01226316 [244]	ER+ AKT1^E17K^-mutant MBC Prior endocrine treatment	Capivasertib + fulvestrant vs. Capivasertib alone	CBR 50% vs. 47% ORR 6% (fulvestrant-pretreated) and 20% (fulvestrant-naïve) vs. 20%
CDK4/6 inhibitors	Palcociclib	PALOMA-1 Phase II NCT00721409 [126]	HR+/HER2- Postmenopausal Advanced BC No prior systemic treatment	Palbocilib + letrozole vs. letrozole alone	PFS 20.2 months vs. 10.2 months (HR 0.488; *p* = 0.0004) PFS 26.1 months vs. 5.7 months (HR 0.299; *p* < 0.0001) in non-Cyclin D1 amplified PFS 18.1 months vs. 11.1 months (HR 0.508; *p* = 0.0046) in Cyclin D1 amplified
		PALOMA-2 Phase III NCT01740427 [245]	HR+/HER2- Postmenopausal Advanced BC No prior systemic treatment	Palbocilib + letrozole vs. placebo + letrozole	PFS 24.8 months vs. 14.5 months (HR 0.58; *p* < 0.001)
		PALOMA-3 Phase III NCT01942135 [246]	HR+/HER2- MBC Prior endocrine therapy	Palbociclib + fulvestrant vs. placebo + fulvestrant	PFS 9.5 months vs. 4.6 months (HR 0.46; *p* < 0.0001)
	Ribociclib	MONALEESA-2 Phase III NCT01958021 [247]	HR+/HER2- Postmenopausal Advanced or MBC	Ribociclib + letrozole vs. placebo + letrozole	PFS 25.3 months vs. 16.0 months (HR 0.568; *p* < 0.0001)
		MONALEESA-3 Phase III NCT02422615 [248]	HR+/HER2- Advanced BC No prior treatment or prior endocrine therapy	Ribociclib + fulvestrant vs. placebo + fulvestrant	PFS 20.5 months vs. 12.8 months (HR 0.593; *p* < 0.001)
	Abemaciclib	MONARCH-2 Phase III NCT02107703 [249]	HR+/HER2- Advanced or MBC Prior endocrine treatment	Abemaciclib + fulvestrant vs. fulvestrant alone	PFS 16.4 months vs. 9.3 months (HR 0.553; *p* < 0.001)
		MONARCH-3 Phase III NCT02246621 [250]	HR+/HER2- Advanced or MBC Prior endocrine treatment	Abemaciclib + anastrozole or letrozole vs. placebo + anastrozole or letrozole	PFS 28.18 months vs. 14.76 months (HR 0.546; *p* < 0.0001)

HR+: hormone receptors positive; HER2-: human epidermal growth factor receptor 2 negative; MBC: metastatic breast cancer; BC: breast cancer; PFS: progression free survival; CBR: clinical benefit rate; ORR: objective response rate; pCR: pathologic complete response; HR: hazard ratio.

**Table 2:** Most recent completed clinical trials on emerging therapies for HER2+ breast cancer.

Targeted Therapy	Drug Name	Trial Number	Patient Population	Trial Arms	Outcomes
Antibodies drug conjugate (ADC)	Trastuzumab-deruxtcan (DS-8201a)	DESTINY-Breast01 Phase II NCT03248492 [264]	HER2+ MBC Prior trastuzumab-emtansine treatment	Trastuzumab-deruxtcan monotherapy	PFS 16.4 months
	Trastuzumab-duocarmycin (SYD985)	Phase I dose-escalation and dose-expansion NCT02277717 [265]	HER2+ Locally advanced or metastatic solid tumors	Trastuzumab-duocarmycin monotherapy	ORR 33%
Modified antibodies	Margetuxumab (MGAH22)	SOPHIA Phase III NCT02492711 [266]	HER2+ Advanced or MBC Prior anti-HER2 therapies	Margetuximab + chemotherapy vs. trastuzumab + chemotherapy	PFS 5.8 months vs. 4.9 months (HR 0.76; *p* = 0.03) OS 21.6 months vs. 19.8 months (HR 0.89; *p* = 0.33) ORR 25% vs. 14% (*p* < 0.001)
Tyrosine kinase inhibitors	Tucatinib	HER2CLIMB Phase II NCT02614794 [267]	HER2+ Locally advanced or MBC Prior anti-HER2 therapies	Tucatinib + trastuzumab and capecitabine vs. placebo + trastuzumab and capecitabine	PFS 33.1% (7.8 months) vs. 12.3% (5.6 months) (HR 0.54; *p* < 0.001) PFS 24.9% vs. 0% (HR 0.48; *p* < 0.001) in brain metastases patients OS 44.9% vs. 26.6% (HR 0.66; *p* = 0.005)
	Poziotinib	NOV120101-203 Phase II NCT02418689 [268]	HER2+ MBC Prior chemotherapy and trastuzumab	Poziotinib monotherapy	PFS 4.04 months
HER2-derived peptide vaccine	E75 (NeuVax)	Phase I/II NCT00841399 NCT00854789 [269]	HER2+ Node-positive or high-risk node-negative BC HLA2/3+	E75 vaccination vs. non-vaccination	DFS 89.7% vs. 80.2% (*p* = 0.008) DFS 94.6% in optimal dosed patients (*p* = 0.005 vs. non-vaccination)
	GP2	Phase II NCT00524277 [270]	HER2 (IHC 1-3+) Disease free Node-positive or high-risk node-negative BC HLA2+	GP2 + GM-CSF vs. GM-CSF alone	DFS 94% vs. 85% (*p* = 0.17) DFS 100% vs. 89% in HER2-IHC3+ (*p* = 0.08)
	AE37	Phase II NCT00524277 [271]	HER2 (IHC 1-3+) Node-positive or high-risk node-negative BC	AE37 + GM-CSF vs. GM-CSF alone	DFS 80.8% vs. 79.5% (*p* = 0.70) DFS 77.2% vs. 65.7% (*p* = 0.21) HER2-low DFS 77.7% vs. 49.0% (*p* = 0.12) TNBC
PI3K inhibitors	Alpelisib	Phase I NCT02167854 [272]	HER2+ MBC with a *PIK3CA* mutation Prior ado-trastuzumab emtansine and pertuzumab	Alpelisib + Trastuzumab + LJM716	Toxicities limited drug delivery 72% for alpelisib 83% for LJM716
		Phase I NCT02038010 [273]	HER2+ MBC Prior trastuzumab-based therapy	Alpelisib + T-DM1	PFS 8.1 months ORR 43% CBR 71% and 60% in prior T-DM1 patients
	Copanlisib	PantHER Phase Ib NCT02705859 [274]	HER2+ Advanced BC Prior anti-HER2 therapies	Copanlisib + trastuzumab	Stable disease 50%
mTOR inhibitors	Everolimus	BOLERO-1 Phase III NCT00876395 [275]	HER2+ Locally advanced BC No prior treatment	Everolimus + trastuzumab vs. placebo + trastuzumab	PFS 14.95 months vs. 14.49 months (HR 0.89; *p* = 0.1166) PFS 20.27 months vs. 13.03 months (HR 0.66; *p* = 0.0049)
		BOLERO-3 Phase III NCT01007942 [276]	HER2+ Advanced BC Trastuzumab-resistant Prior taxane therapy	Everolimus + trastuzumab and vinorelbine vs. placebo + trastuzumab and vinorelbine	PFS 7.00 months vs. 5.78 months (HR 0.78; *p* = 0.0067)
CDK4/6 inhibitors	Palbociclib	SOLTI-1303 PATRICIA Phase II NCT02448420 [277]	HER2+ ER+ or ER- MBC Prior standard therapy including trastuzumab	Palbociclib + trastuzumab	PFS 10.6 months (luminal) vs. 4.2 months (non-luminal) (HR 0.40; *p* = 0.003)
	Ribociclib	Phase Ib/II NCT02657343 [278]	HER2+ Advanced BC Prior treatment with trastuzumab, pertuzumab, and trastuzumab emtansine	Ribociclib + trastuzumab	PFS 1.33 months No dose-limiting toxicities
	Abemaciclib	MonarcHER Phase II NCT02675231 [279]	HER2+ Locally advanced or MBC Prior anti-HER2 therapies	Abemaciclib + trastuzumab and fulvestrant (A) vs. abemaciclib + trastuzumab (B) vs. standard-of-care chemotherapy + trastuzumab (C)	PFS 8.3 months (A) vs. 5.7 months (C) (HR 0.67; *p* = 0.051) PFS 5.7 months (B) vs. 5.7 months (C) (HR 0.97; *p* = 0.77)

HER2+: human epidermal growth factor receptor 2 positive; ER+: estrogen receptor positive; HLA2/3: human leucocyte antigen 2/3; MBC: metastatic breast cancer; BC: breast cancer; PFS: progression free survival; CBR: clinical benefit rate; ORR: objective response rate; DFS: disease-free survival OS: overall survival GM-CSF: granulocyte macrophage colony-stimulated factor; HR: hazard ratio.

**Table 3:** Most recent completed clinical trials on emerging therapies for TNBC.

Targeted Therapy	Drug Name	Trial Number	Patient Population	Trial Arms	Outcomes
Antibodies Drug Conjugate	Sacituzumab govitecan	ASCENT Phase III NCT02574455 [309]	TNBC MBC Prior standard treatment	Sacituzumab govitecan vs. single-agent chemotherapy	PFS 5.6 months vs. 1.7 months (HR 0.41; *p* < 0.001) PFS 12.1 months vs. 6.7 months (HR 0.48; *p* < 0.001)
VEGF inhibitors	Bevacizumab	BEATRICE Phase III NCT00528567 [310]	Early TNBC Surgery	Bevacizumab + chemotherapy vs. chemotherapy alone	IDFS 80% vs. 77% OS 88% vs. 88%
		CALGB 40603 Phase II NCT00861705 [311]	TNBC Stage II to III	Bevacizumab + chemotherapy vs. chemotherapy alone or Carboplatin + chemotherapy vs. chemotherapy alone	pCR 59% vs. 48% (*p* = 0.0089) (Bevacizumab) pCR 60% vs. 44% (*p* = 0.0018) (Carboplatin)
EGFR inhibitors	Cetuximab	TBCRC 001 Phase II NCT00232505 [312]	TNBC MBC	Cetuximab + carboplatin	Response < 20% TTP 2.1 months
		Phase II NCT00463788 [313]	TNBC MBC Prior chemotherapy treatment	Cetuximab + cisplatin vs. cisplatin alone	ORR 20% vs. 10% (*p* = 0.11) PFS 3.7 months vs. 1.7 months (HR 0.67; *p* = 0.032) OS 12.9 months vs. 9.4 months (HR 0.82; *p* = 0.31)
mTORC1 inhibitors	Everolimus	Phase II NCT00930930 [314]	TNBC Stage II or III Neoadjuvant treatment	Everolimus + cisplatin and paclitaxel vs. placebo + cisplatin and paclitaxel	pCR 36% vs. 49%
Akt inhibitors	Ipatasertib	LOTUS Phase II NCT02162719 [315]	TNBC Locally advanced or MBC No prior sytemic therapy	Ipatasertib + paclitaxel vs. placebo + paclitaxel	PFS 6.2 months vs. 4.9 months (HR 0.60; *p* = 0.037) PFS 6.2 months vs. 3.7 moths (HR 0.58; *p* = 0.18) in PTEN-low patients
		FAIRLANE Phase II NCT02301988 [316]	Early TNBC Neoadjuvant treatment	Ipatasertib + paclitaxel vs. placebo + paclitaxel	pCR 17% vs. 13% pCR 16% vs. 13% PTEN-low patients pCR 18% vs. 12% PIK3CA/AKT1/PTEN-altered patients
	Capivasertib	PAKT Phase II NCT02423603 [317]	TNBC MBC No prior chemotherapy treatment	Capivasertib + paclitaxel vs. placebo + paclitaxel	PFS 5.9 months vs. 12.6 months (HR 0.61; *p* = 0.04)
Androgen receptor inhibitors	Bicalutamide	Phase II NCT00468715 [318]	HR- AR+ or AR- MBC	Bicalutamide monotherapy	CBR 19% PFS 12 weeks
	Enzalutamide	Phase II NCT01889238 [319]	TNBC AR+ Locally advanced or MBC	Enzalutamide monotherapy	CBR 25% OS 12.7 months
CYP17 inhibitors	Abiraterone acetate	UCBG 12-1 Phase II NCT01842321 [320]	TNBC AR+ Locally advanced or MBC Centrally reviewed Prior chemotherapy	Abiraterone acetate + prednisone	CBR 20% ORR 6.7% PFS 2.8 months
Anti-PDL1 antibodies	Atezolizumab	Impassion 130 Phase III NCT02425891 [321]	TNBC Locally advanced or MBC No prior treatment	Atezolizumab + nab-paclitaxel vs. placebo + nab-paclitaxel	OS 21.0 months vs. 18.7 months (HR 0.86; *p* = 0.078) OS 25.0 months vs. 18.0 months (HR 0.71, 95% CI 0.54–0.94)) in PDL-1+ patients
		Impassion 031 Phase III NCT03197935 [322]	TNBC Stage II to III No prior treatment	Atezolizumab + chemotherapy vs. placebo + chemotherapy	pCR 95% vs. 69% *p* = 0.0044
	Durvalumab	GeparNuevo Phase II NCT02685059 [323]	TNBC MBC Stromal tumor-infiltrating lymphocyte (sTILs)	Durvalumab vs. placebo	pCR 53.4% vs. 44.2% pCR 61.0% vs. 41.4% in window cohort
		SAFIRO BREAST-IMMUNO Phase II NCT02299999 [324]	HER2- MBC Prior chemotherapy	Durvalumab vs. maintenance chemotherapy	HR of death 0.37 for PDL-1+ patients HR of death 0.49 for PDL-1- patients
		Phase I NCT02484404 [325]	Recurrent women’s cancers including TNBC	Durvalumab + cediranib + olaparib	Partial response 44% CBR 67%
	Avelumab	JAVELIN Phase Ib NCT01772004 [326]	MBC Prior standard-of-care therapy	Avelumab monotherapy	ORR 3.0% overall ORR 5.2% in TNBC ORR 16.7% in PDL-1+ vs. 1.6% in PDL-1- overall ORR 22.2.% in PDL-1+ vs. 2.6% in PDL-1- in TNBC
Anti-PD1 antibodies	Pembrolizumab	KEYNOTE-086 Phase II NCT02447003 [327]	TNBC MBC Prior or no prior systemic therapy	Pembrolizumab monotherapy	Previously treated patients: ORR 5.3% overall ORR 5.7% PDL-1+ patients PFS 2.0 months OS 9.0 months Non-previously pretreated: ORR 21.4% PFS 2.1 months OS 18.0 months
		KEYNOTE-119 Phase III NCT02555657 [328]	TNBC MBC Prior systemic therapy	Pembrolizumab vs. chemotherapy	OS 12.7 months vs. 11.6 months (HR 0.78; *p* = 0.057) in PDL1+ patients OS 9.9 months vs. 10.8 months (HR 0.97, 95% CI 0.81–1.15)
		KEYNOTE-355 Phase III NCT02819518 [329]	TNBC MBC No prior systemic therapy	Pembrolizumab + chemotherapy vs. placebo + chemotherapy	PFS 9.7 months vs. 5.6 months (HR 0.65; *p* = 0.0012) in PDL-1+ patients PFS 7.6 months vs. 5.6 months (HR 0.74; *p* = 0.0014)
		KEYNOTE-522 Phase III NCT03036488 [330]	Early TNBC Stage II to III No prior treatment	Pembrolizumab + paclitaxel and carboplatin vs. placebo + paclitaxel and carboplatin	pCR 64.8% vs. 51.2 % (*p* < 0.001)
Anti-CDL4 antibodies	Tremelimumab	Phase I [331]	Incurable MBC	Tremelimumab + radiotherapy	OS 50.8 months
Vaccines	PPV	Phase II UMIN000001844 [332]	TNBC MBC Prior systemic therapy	PPV vaccine	PFS 7.5 months OS 11.1 months
	STn-KLH	Phase III NCT00003638 [333]	MBC Prior chemotherapy Partial or complete response	STn-KLH vaccine vs. non-vaccine	TTP 3.4 months vs. 3.0 months

TNBC: triple negative breast cancer; HER2: human epidermal growth factor receptor; HR: hormonal receptor; MBC: metastatic breast cancer; BC: breast cancer; AR: androgen receptor; PPV: personalized peptide vaccine; PFS: progression free survival; CBR: clinical benefit rate; ORR: objective response rate; IDFS: invasive disease-free survival; OS: overall survival; TTP: time to progression; pCR: pathologic complete response; HR: hazard ratio.

**Table 4:** Ongoing clinical trials on emerging therapies for BC treatment for all BC molecular subtypes.

Targeted Therapy	Drug Name	Patient Population	Trial Arms	Outcome Measures	Status	Trial
PI3K inhibitors	Copanlisib	HR+/HER2- Postmenopausal Invasive BC Stage I to IV	Copanlisib + letrozole and palbocilib vs. copanlisib + letrozole vs. letrozole + palbociclib	pCR ORR DLT	Active, not recruiting	Phase I/II NCT03128619
		HR+/HER2- MBC Stage IV	Copanlisib + fulvestrant vs. fulverstant alone	PFS ORR	Recruiting	Phase I/II NCT03803761
		HER2+ PIK3CA or PTEN mutated MBC Stage IV	Copanlisib + trastuzumab + pertuzumab vs. trastuzumab + pertuzumab	PFS OS DLT	Recruiting	Phase Ib/II NCT04108858
		TNBC MBC Unresectable BC Stage III to IV	Copanlisib + eribulin vs. eribulin alone	MTD PFS ORR CBR	Recruiting	Phase I/II NCT04345913
	Taselisib	HER2+ MBC Recurrent BC	Taselisib + TDM-1 vs. taselisib + TDM-1 and pertuzumab vs. taselisib + pertuzumab and trastuzumab vs. taselisib + pertuzumab and trastuzumab and paclitaxel	MTD PFS CBR	Active, not recruiting	Phase Ib NCT02390427
mTOR inhibitors	Everolimus	TNBC Advanced BC Prior systemic treatment	Everolimus + caroboplatin vs. carboplatin alone	PFS ORR OS CBR	Recruiting	Phase II NCT02531932
Akt inhibitors	Capivasertib	HR+/HER2- Locally advanced or MBC Prior systemic treatment	Capivasertib + palbociclib and fulvesrant vs. pplacebo + palbociclib and fulvesrant	DLT PFS ORR CBR OS	Recruiting	Phase Ib/III NCT04862663
		HR+/HER2- Locally advanced or MBC Prior systemic treatment	Capivasertib + fulvesrant vs. pplacebo + fulvesrant	PFS ORR CBR OS	Recruiting	Phase III NCT04305496
		TNBC Locally advanced or MBC No prior systemic treatment	Capivasertib + paclitaxel vs. placebo + paclitaxel	PFS ORR CBR OS	Recruiting	Phase III NCT03997123
	Ipatasertib	ER+/HER2- Post-menopausal Prior CDK4/6 inhibitors and AIs	Ipatasertib + fulvestrant verus placebo + fulvestrant	PFS ORR CBR OS	Recruiting	Phase III NCT04650581
		HR+/HER2- Post-menopausal Locally advanced or MBC Prior systemic treatment	Ipatasertib + fulverstrant vs. ipatasertib + AI vs. ipatasertib + fulvestrant and palbociclib	PFS ORR OS	Recruiting	Phase III NCT03959891
		HER2+ PIK3CA mutated Locally advanced or MBC Prior systemic treatment	Ipatasertib + trastuzumab and pertuzumab	Safety and tolerability PFS ORR CBR	Recruiting	Phase Ib NCT04253561
		TNBC MBC Stage IV No prior treatment	Ipatasertib + carboplatin and paclitaxel vs. ipatasertib + carboplatin vs. ipatasertib + capecitabine and atezolizumab	PFS CBR OS TTF	Recruiting	Phase I/Ib NCT03853707
		TNBC Locally advanced or MBC Prior systemic treatment	Ipatasertib + capecitabine vs. ipatasertib + eribulin vs. ipatasertib + carboplatin and gemcitabine	PFS ORR CBR OS TTR	Recruiting	Phase IIa NCT04464174
CDK4/6 inhibitors	Ribociclib	HR+/HER2- PIK3CA mutated Postmenopausal Locally advanced or MBC No prior systemic treatment	Ribociclib + letrozole	TTP CBR	Active, not recruiting	Phase III NCT03439046
		HR+/HER2- MBC Prior systemic treatment	Ribociclib + (anti-hormonal treatment) anastrozole and exemestane and letrozole and fulvestrant vs. anti-hormonal treatment alone	PFS CBR OS	Recruiting	Phase II NCT03913234
		HR+/HER2- Early BC No prior endocrine therapy	Ribociclib + endocrine therapy vs. endocrine therapy alone	IDFS RFS DDFS OS	Recruiting	Phase III NCT03701334
		HR+/HER2- Locally advanced or MBC No prior systemic treatment	Ribociclib monotherapy	ORR PFS CBR TTP	Active, not recruiting	Phase II NCT03822468
		HR+/HER2+ Postmenopausal Locally advanced or MBC No prior systemic treatment	Ribociclib + trastuzumab + letrozole	PFS OS	Recruiting	Phase Ib/II NCT03913234
		HER2+ Locally advanced or MBC Prior systemic treatment	Ribociclib monotherapy	MTD PFS ORR CBR OS	Active, not recruiting	Phase Ib/II NCT02657343
		HER2- Locally advanced or MBC Prior chemotherapy treatment	Ribociclib + capecitabine	MTD Safety Efficacy	Recruiting	Phase I dose-escalation NCT02754011
		TNBC AR+ MBC or unresectable BC Prior systemic treatment	Ribociclib monotherapy	MTD PFS ORR CBR OS	Active, not recruiting	Phase I/II NCT03090165
	Abemaciclib	HR+/HER2- Post-menopausal Stage I to III Prior endocrine treatment	Abemaciclib + fulvestrant	pCR ORR RFS	Recruiting	Phase II NCT04305236
		HR+/HER2- Stage II to III No prior systemic treatment	Abemaciclib + letrozole	iEFS CR	Recruiting	Phase II NCT04293393
		HR+/HER2- Locally advanced or MBC Nor prior systemic treatment	Abemaciclib + AIs	ORR CBR TTP DoCB	Recruiting	Phase II NCT04227327
		HER2+ Locally advanced or MBC Prior systemic treatment	Abemaciclib + TDM-1 vs. TDM-1 alone	ORR OS	Recruiting	Phase II NCT04351230
		TNBC Rb+ Locally advanced or MBC Prior chemotherapy treatment	Abemaciclib monotherapy	ORR PFS OS CBR	Recruiting	Phase II NCT03130439
	Palbociclib	HR+/HER2- Post-menopausal Locally advanced or MBC Prior chemotherapy treatment	Palbociclib + fulvestrant	PFS ORR CBR OS	Recruiting	Phase II NCT04318223
		ER+ Stage I to III No prior systemic treatment	Palbociclib + endocrine therapy vs. endocrine therapy alone	pCR Safety Tolerability	Recruiting	Phase I NCT03573648
		ER+/HER2+ MBC Prior systemic treatment	Palbociclib + letrozole and TDM-1	ORR CR SD	Active, not recruiting	Phase I/II NCT03709082
		HER2+ Post-menopausal MBC No prior systemic treatment	Palbociclib + anastrozole + trastuzumab + pertuzumab	DLT MTD CBR PFS	Recruiting	Phase I/II NCT03304080
		HER2+ Rb+ MBC Prior anti-HER2 treatment	Palbociclib + TDM-1	MTD DLT	Active, not recruiting	Phase Ib NCT01976169
Antibodies drug conjugates	Trastuzumab-deruxtcan	HER2+ Unresectable or MBC Prior TDM-1 treatment	Trastuzumab-deruxtcan vs. trastuzumab + capecitabine vs. lapatinib + capecitabine	PFS OS ORR DoR	Active, not recruiting	Phase III NCT03523585
		HER2+ Unresectable or MBC Prior anti-HER2 treatment	Trastuzumab-deruxtcan vs. TDM-1	PFS OS ORR DoR	Active, not recruiting	Phase III NCT03529110
		HER2- Unresectable or MBC Prior systemic treatment	Trastuzumab-deruxtcan vs. chemotherapy	PFS OS ORR DoR	Active, not recruiting	Phase III NCT03734029
	Trastuzumab-duocarmycin	HER2+ Locally advanced or MBC Prior anti-HER2 treatment	Trastuzumab-duocarmycin vs. standard treatment	PFS OS ORR	Active, not recruiting	Phase III NCT03262935
	RC48	HER2+ Locally advanced or MBC Prior systemic treatment	RC48 vs. lapatinib + capecitabine	PFS ORR DoR CBR OS	Recruiting	Phase II NCT03500380
		HER2+ or HER2- Locally advanced or MBC No prior systemic treatment	RC48 monotherapy	ORR CBR PFS	Recruiting	Phase Ib NCT03052634
	PF06804103	HER2+ or HER2- Solid tumors	PF06804103 alone vs. PF06804103 + letrozole and palbociclib	DLT PFS TTP	Recruiting	Phase I dose-escalation NCT03284723
	Ladiratuzumab vedotin	TNBC Locally advanced or MBC No prior chemotherapy	Ladiratuzumab vedotin monotherapy	DLT ORR DoR PFS OS	Recruiting	Phase I NCT01969643
Bispecific antibodies	MCLA-128	HER2+ or ER+/HER2- Locally advanced or MBC No prior systemic treatment	MCLA-128 + trastuzumab vs. MCLA-128 + trastuzumab and vinorelbine or MCLA-128 + endocrine therapy	CBR PFS ORR DoR OS	Active, not recruiting	Phase II NCT03321981
	ZW25 (Zanidatamab)	HR+/HER2+ Locally advanced or MBC Prior anti-HER2 treatment	ZW25 + Palbociclib + fulvestrant	DLT PFS IAEs	Recruiting	Phase IIa NCT04224272
	ISB 1302	HER2+ MBC Prior anti-HER2 treatment	ISB 1302 monotherapy	MTD IAEs	Terminated	Phase I/II NCT03983395
	PRS-343	HER2+ solid tumors No prior systemic treatment	PRS-343 + atezolizumab	DLT ORR DoR CR IAEs	Active, not recruiting	Phase Ib NCT03650348
		HER2+ solid tumors Locally advanced or MBC	PRS-343 monotherapy	IAEs	Recruiting	Phase I NCT03330561
Androgen receptor inhibitors	Bicalutamide	TNBC AR+ Locally advanced or MBC	Bicalutamide alone vs. chemotherapy	PFS CBR ORR OS	Terminated	Phase III NCT03055312
		TNBC AR+ Unresectable or MBC Up to one prior systemic treatment	Bicalutamide + ribociclib	MTD CBR ORR PFS OS	Active, not recruiting	Phase I/II NCT03090165
		TNBC or HER2+ AR+ Stage IV MBC Prior systemic treatment	Bicalutamide monotherapy	pCR PFS Safety	Active, not recruiting	Phase II NCT00468715
		TNBC or ER+ AR+ MBC Prior systemic treatment	Bicalutamide + Palbociclib	PFS CBR Safety Tolerability	Active, not recruiting	Phase I/II NCT02605486
	Enzalutamide	TNBC AR+ Stage I to III No prior treatment	Enzalutamide + paclitaxel	pCR PFS	Recruiting	Phase IIb NCT02689427
		TNBC AR+ PTEN+ Stage III to IV MBC No prior treatment	Enzalutamide + alpelisib	MTD PFS CBR	Recruiting	Phase Ib NCT03207529
		TNBC AR+ Stage I to III Prior chemotherapy treatment	Enzalutamide monotherapy	TDR	Active, not recruiting	Feasibility study NCT02750358
	CR1447	ER+ or TNBC AR+ MBC One prior systemic treatment	CR1447 monotherapy	CR PR SD	Active, not recruiting	Phase II NCT02067741
Anti-PD1 antibodies	Pembrolizumab	HR+/HER2- Locally advanced or MBC Prior chemotherapy and CDK4/6 inhibitors treatments	Pembrolizumab + paclitaxel	ORR CBR PFS DoR OS	Recruiting	Phase II NCT04251169
		HER2+ MBC Prior systemic treatment No prior TDM-1 treatment	Pembrolizumab + TDM-1	ORR PFS DoR OS	Active, recruiting	Phase Ib NCT03032107
		HR+/HER2- MBC Prior systemic treatment	Pembrolizumab + fulvestrant	ORR PFS	Recruiting	Phase II NCT03393845
		HR+ or TNBC MBC Prior systemic treatment	Pembrolizumab + Nab-paclitaxel	ORR PFS OS	Recruiting	Phase II NCT02752685
		TNBC Prior systemic treatment	Pembrolizumab + cyclophosphamide	PFS	Active, recruiting	Phase II NCT02768701
		TNBC MBC Prior systemic treatment	Pembrolizumab + Carboplatin and Nab-paclitaxel	PFS DCR	Active, recruiting	Pilot study NCT03121352
		TNBC or ER+ or HER2+ BRCA mutated Locally advanced or MBC Prior systemic treatment	Pembrolizumab + olaparib	ORR PFS OS CBR DoR	Recruiting	Phase II NCT03025035
Anti-CTLA-4 antibodies	Tremelimumab	HR+/HER2- Stage I to III No prior systemic treatment	Tremelimumab + durvalumab	IAEs pCR	Active, not recruiting	Pilot study NCT03132467
HER2-derived vaccines	E75	HER2+ Stage I to III Prior systemic treatment	E75 vaccine + trastuzumab vs. trastuzumab + GM-CSF	DFS RFS	Active, not recruiting	Phase II NCT02297698
	GP2	HER2+ Prior systemic treatment except for trastuzumab	G2P vaccine + GM-SCF and trastuzumab vs. trastuzumab	IAEs	Active, not recruiting	Phase Ib NCT03014076
	AE37	TNBC Prior systemic treament	AE37 vaccine + pembrolizumab	ORR PFS OS CBR	Active, not recruiting	Phase II NCT04024800
Other vaccines	PVX-140	TNBC HLA-2+ Stage II or III Prior systemic treatment	PVX-140 + durvalumab	DLT DFS IAEs	Active, not recruiting	Phase Ib NCT02826434
	Neoantigen DNA vaccine	TNBC Post-menopausal Prior systemic treatment	Neoantigen DNA vaccine + durvalumab vs. Neoantigen DNA vaccine alone	Safety Immune response	Recruiting	Phase I NCT03199040
	Dendritic cell vaccine	TNBC or ER+/HER2- Locally advanced	DC vaccine + chemotherapy	Safety pCR DFS	Completed	Pilot study NCT02018458

TNBC: triple negative breast cancer; HER2: human epidermal growth factor receptor 2; ER: estrogen receptor; MBC: metastatic breast cancer; BC: breast cancer; HR: hormonal receptor; PFS: progression free survival; CBR: clinical benefit rate; ORR: objective response rate; DFS: disease-free survival; OS: overall survival; TTP: time to progression. pCR: pathologic complete response; GM-CSF: granulocyte macrophage colony-stimulated factor; DLT: dose-limiting toxicities; MTD: maximum tolerated dose; TTF: time to treatment failure; TTR: time to treatment response; iDFS: invasive disease-free survival; RFS: recurrence free survival; DDFS: distant disease-free survival; iEFS: invasive events-free survival; CR: clinical response; DoCB: duration of clinical benefit; SD: stable disease; DoR: duration of response; IAEs: incidence of adverse events; TDR: treatment discontinuation rate; PR: partial response; DCR: disease control rate; HR: hazard ratio.

## Data Availability

The study did not report any data.

## Abbreviation

ABC	ATP binding cassette
ADC	antibody-drug conjugate
ADCC	antibody dependent cell cytotoxicity
AI	aromatase inhibitor
AIB1	amplified in breast cancer 1
ALND	axillary lymph node dissection
AR	androgen receptor
ATM	ataxia-telangiesctasia mutated
BC	breast cancer
BCRP	breast cancer resistant protein
BRCA	breast cancer gene
BsAb	bispecific antibody
CBR	clinical benefice rate
CDK4/6	cyclin-dependent kinase
CR	clinical response
CSC	cancer stem cell
CTLA4	cytotoxic T lymphocyte-associated protein 4
DDFS	distant disease-free survival
DFS	disease-free survival
DLT	dose-limiting toxicities
DoCB	duration of clinical benefit
DoR	duration of response
EGF	epidermal growth factor
EGFR	epidermal growth factor receptor
ER	estrogen receptor
FDA	food and drug administration
gBRCAm	germline BRCA mutation
HB-EGF	heparin-binding EGF-like growth factor
HER2	human epidermal growth factor receptor 2
HGF	hepatocyte growth factor
HIF1-α	hypoxia-inducible factor 1 alpha
HR	hormone receptor
HR	hazard ratio
IAES	incidence of adverse events
IDFS	invasive disease-free survival
iEFS	invasive events-free survival
IGF-1	insulin growth factor 1
IGF-1R	insulin growth factor receptor 1
MAP	microtubule associated protein
MAPK	mitogen activated protein kinase
MBC	metastatic breast cancer
MTD	maximum tolerated dose
mTOR	mammalian target of rapamycin
NAC	neoadjuvant chemotherapy
ORR	overall response rate
OS	overall survival
PARP	poly-(ADP-ribose) polymerase protein
PARPi	poly-(ADP-ribose) polymerase protein inhibitor
pCR	predicted complete response
PD-1	programmed cell death protein receptor
PDL-1	programmed cell death protein ligand
PFS	progression-free survival
PI3K	phosphoinositide 3-kinase
PPV	personalized peptide vaccine
PR	progesterone receptor
PR	partial response
PTEN	phosphatase and tensin homolog
Ras-ERK	extracellular-signal-regulated kinase
RFS	recurrence-free survival
SD	stable disease
SERD	selective estrogen receptor degrader
SERM	selective estrogen receptor modulator
SLNB	sentinel lymph mode biopsy
STnKLH	sialyl-TN keyhole limpet hemocyanin
T-DM1	trastuzumab-emtansine
TKI	tyrosine kinase inhibitor
TNBC	triple-negative breast cancer
Trop2	trophoblast antigen 2
TTF	time to treatment failure
TTP	time to treatment progression
TTR	time to treatment response
VEGF	vascular endothelial growth factor
